# Intra-locked G-quadruplex structures formed by irregular DNA G-rich motifs

**DOI:** 10.1093/nar/gkaa008

**Published:** 2020-02-26

**Authors:** Arijit Maity, Fernaldo Richtia Winnerdy, Weili Denyse Chang, Gang Chen, Anh Tuân Phan

**Affiliations:** 1 School of Physical and Mathematical Sciences, Nanyang Technological University, Singapore 637371, Singapore; 2 School of Biological Sciences, Nanyang Technological University, Singapore 637551, Singapore; 3 NTU Institute of Structural Biology, Nanyang Technological University, Singapore 636921, Singapore

## Abstract

G-rich DNA sequences with tracts of three or more continuous guanines (G_≥3_) are known to have high propensity to adopt stable G-quadruplex (G4) structures. Bioinformatic analyses suggest high prevalence of G-rich sequences with short G-tracts (G_≤2_) in the human genome. However, due to limited structural studies, the folding principles of such sequences remain largely unexplored and hence poorly understood. Here, we present the solution NMR structure of a sequence named *AT26* consisting of irregularly spaced G_2_ tracts and two isolated single guanines. The structure is a four-layered G4 featuring two bi-layered blocks, locked between themselves in an unprecedented fashion making it a stable scaffold. In addition to edgewise and propeller-type loops, *AT26* also harbors two V-shaped loops: a 2-nt V-shaped loop spanning two G-tetrad layers and a 0-nt V-shaped loop spanning three G-tetrad layers, which are named as V_S_- and V_R_-loop respectively, based on their distinct structural features. The intra-lock motif can be a basis for extending the G-tetrad core and a very stable intra-locked G4 can be formed by a sequence with G-tracts of various lengths including several G_2_ tracts. Findings from this study will aid in understanding the folding of G4 topologies from sequences containing irregularly spaced multiple short G-tracts.

## INTRODUCTION

Guanine-rich nucleic acids are known to have high propensity to fold into a non-canonical secondary structure, consisting of planar G•G•G•G tetrads (1), termed G-quadruplex (G4) (2). In the last three decades, G4 structures have attracted increasing attention, given that G-rich sequences are found in biologically relevant sites, such as telomeres, minisatellites, promoters, immunoglobulin class switch regions, replication initiation sites, mRNA untranslated regions (UTRs) and introns (3). G4 structures were detected in the cells (4) and were revealed to play important roles in regulating cellular processes, such as replication, transcription, splicing and translation (5). G4s are potential targets in diseases, such as cancers (6,7), ALS/FTD (8,9) and fragile X syndrome (10). On the other hand, G4-forming sequences have been shown to exhibit anti-cancer (11–14), anti-coagulant (15,16) and anti-HIV properties (17–19). G4 structures are highly polymorphic with regards to molecularity, relative strand arrangement, loop architecture and glycosidic bond conformation of the guanines (20–22). The stability of G4s generally increases with the increasing number of stacked G-tetrads (23), but shares an inverse relationship with the loop lengths (24–28). The majority of G4 structures reported in literature consists of three G-tetrad layers, with few reported structures containing either two layers, four layers or more (29).

Several computational algorithms (e.g. Quadparser, G4P calculator) (30–32) were developed to predict the presence of potential G4-forming sequences in genomic databases. They generally considered stretches of three or more guanines separated by loops of sizes up to seven nucleotides (G_3+_N_1–7_G_3+_N_1–7_G_3+_N_1–7_G_3+_) for the formation of stable G4 structures, resulting in ∼380,000 predicted G4-forming sequences in the human genome (31). However, over the past decade, there were numerous examples of G4 structures that did not necessarily obey the general sequence scheme, such as G4 structures with bulges (33), missing guanines (34–36), extremely long loops (up to 70 nt) (28,37,38) and duplex-containing loops (39–45). In addition, the inclusions of novel types of assemblies in quadruplex structures, such as stacking base triads (46), non-canonical tetrads (47–50), pentads (51), hexads (52), heptads (53) and octads (54), were not accounted for in the general algorithm. G4-forming potential of sequences with shorter G-stretches (G_≤2_) were also not considered. A different algorithmic approach, which considered further factors like G-richness and G-skewness, showed that the number of potential G4-forming sequences in the human genome could be up to 10-fold higher than predicted earlier (55). Recent *in vitro* experimental studies have found over 700,000 G4-forming sequences in the human genome, out of which ∼450,000 were not detected by the general algorithm (56). In a chromatin context, much fewer G4-forming motifs were detected, which appeared mostly in the regulatory and nucleosome-depleted regions, showing the effect of the cell state on the G4 formation (57).

Bioinformatic analyses have shown that sequences with G_2_ tracts are abundant in the human genome (58). G_2_ tract containing GGX or XGG tri-nucleotide repeat (TNR) sequences were found to be condensed in specific sites of the genome and have biological relevance (58). For example, CGG repeats occurring at the 5′-UTR of *FMR1* gene is associated with diseases, such as fragile X syndrome (FXS) and fragile X-associated tremor ataxia syndrome (FXTAS) (59), while recent reports have linked TGG repeats mediated microdeletion at human chromosome 14q32.2 with Kagami-Ogata syndrome (60,61). Some of the TNRs were revealed to form diverse types of G4s (62,63), while other studies exemplified the G4-forming potential of sequences containing G_2_ tracts other than TNRs (64–67). These works show that sequences containing multiple G_2_ tracts are capable of forming stable G4 structures. However, due to limited number of structural studies, the folding principles of G4 structures from sequences consisting of multiple G_2_ tracts remain poorly understood to date.

An anti-cancer DNA aptamer containing TGG repeats (known as *AGRO100* or *AS1411*) was shown to adopt a mixture of various G4 structures in solution (68). The G4 structures of three *AGRO100* derivates, namely *AT11*, d[(TGG)_3_TTGTTG(TGG)_4_T], *AT27* d[(TGG)_4_TTG(TGG)_3_TGTT] and *AT21* d[(TGG)_4_TTG(TGG)TGT(TGG)_2_T], were resolved (69–71). These three derivatives adopt exceptionally different structures despite their very similar sequences, providing an excellent platform to study the intricacy of irregular G4-forming sequences without long G_≥3_ tracts.

Herein, we studied a 28-mer G-rich sequence *AT26*, d[(TGG)_4_TTG(TGG)_3_TTGT], which includes a total of 16 guanines, distributed into multiple G_2_ tracts and two isolated single guanines. It has the same composition as *AT11*, *AT21* and *AT27*, differing only in the position of the isolated guanines, and yet its structure is found to be dramatically different from the other three, resonating a previous observation where a slight change in a G4-forming sequence dramatically altered its folding topology (72). *AT26* formed a stable four-layered G4 structure, featuring an unprecedented folding topology. It adopts a novel *intra-locked* conformation, whereby the overall four-layered G4 consists of two bi-layered blocks with three connecting linkers locking them. The discovery would benefit towards understanding the folding principles of G4 structures by sequences consisting of multiple short G-tracts.

## MATERIALS AND METHODS

### Sample preparation

Unlabeled DNA oligonucleotides were purchased from IDT in Singapore with standard desalting purification protocol. Sample purity, measured with ESI-MS, was >99%. All site-specifically labeled DNA oligonucleotides were chemically synthesized in-house on an ABI 394 DNA synthesizer using phosphoramidites from Glen Research and Cambridge Isotope Laboratories. Purification protocol from Glen research was followed to purify them. The purified DNA oligonucleotides were dialyzed successively against water, 10 mM KCl, and water again. The samples were frozen, lyophilized, and dissolved in 20 mM KPi buffer at pH 7.0 containing 120 mM KCl. DNA concentration was calculated in terms of strand molarity using the Beer-Lambert law (*A* = *ϵlc*, where *A*, *ϵ*, *l* and *c* stand for absorbance, extinction coefficient, pathlength of light and concentration of the solution, respectively). Pathlength of the light was 1 cm, the extinction coefficient of the unfolded species was obtained from nearest neighbor approximation. The samples were heated at ∼95°C for 5 min followed by slowly cooling it down to room temperature prior to performing any spectroscopic measurements.

### Circular dichroism spectroscopy

Circular dichroism (CD) spectra were recorded at 20°C on a JASCO-815 spectropolarimeter using 1-cm path length quartz cuvettes and a reaction volume of 500 μl. DNA samples with concentrations of 3–8 μM were dissolved in a 20 mM KPi buffer at pH 7.0 supplemented with 120 mM KCl. Scan range was set to 220–320 nm; scans were performed with 100 nm/min speed, 1-nm data pitch, 1-nm bandwidth and 1 second digital integration time (D.I.T.). An average of five scans was taken for each measurement, the spectral contribution of the buffer was subtracted, and the data were zero-corrected at 320 nm. Molar ellipticity of CD spectra was calculated using the DNA concentration derived from the sample absorbance at 260 nm and the sample extinction coefficient calculated at 260 nm.

The thermal stabilities of the samples were assessed using CD melting. The CD signal at 260 nm was recorded in the temperature range 15–90°C or 15–95°C (only for *AT26SE*), using a temperature ramp rate of 0.2°C/min, sampling interval of 0.5°C, and a digital integration time of 2 s. The melting curves were fit to a two-state model. Two baselines were drawn at the lowest and highest temperatures which corresponded to fully folded and fully unfolded states respectively. The melting temperature was the temperature at which the sample was 50% folded or unfolded. *T*_m_ data was calculated for both unfolding (i.e. while increasing the temperature) and refolding event (while decreasing the temperature). Data reported in Table 3 are the mean and mean deviation of *T*_m_ from the unfolding and refolding process.

### NMR spectroscopy

NMR experiments were performed at 25°C on Bruker Avance II and III spectrometers operating at 600 and 800 MHz, respectively. The DNA concentration for NMR experiments was typically 0.1−1.5 mM in 120 mM KCl, 20 mM KPi (pH 7). Assignment of the imino protons of guanine residues was obtained by ^15^N-filtered experiments using 2% site-specific labeled samples. Assignment of guanine and thymine aromatic protons was obtained by ^15^N or ^13^C filtered experiments using 2% or 4% site-specific labeled samples. Spectra analyses were performed using the Topspin 3.5 (Bruker) and SPARKY 3.1 software (73).

### NMR structure calculation

*NOE distance restraints*. Inter-proton distances for *AT26* were obtained from NOESY experiments performed in H_2_O and D_2_O at various mixing times (100, 200 and 300 ms). For non-exchangeable protons, the peaks were classified as strong, medium, medium-weak and weak corresponding to the distance restraints of (2.7 ± 0.8), (3.8 ± 0.9), (4.6 ± 1.2) and (5.5 ± 1.7) Å respectively. Distances from exchangeable protons were classified as strong, medium and weak corresponding to the distance restraints of (4.0 ± 1.2), (4.8 ± 1.4) and (5.5 ± 1.7) Å respectively. Distances involving thymine methyl protons were altered to be directed toward the methyl carbon with a 0.5 Å looser restraints as compensation.

#### Dihedral restraints

Dihedral angle restraints were imposed to the dihedral angle formed by O4′–C1′–N9–C4 of guanine residues. *Anti*-guanine residues were restricted to an angle of (240 ± 70)°, while *syn*-guanine residues were restricted to an angle of (60 ± 70)°. Dihedral angle restraint was not applied to the G3 residue.

#### Hydrogen-bond restraints

Hoogsteen hydrogen bonds between guanines were restrained using H21–N7, N2–N7, H1–O6 and N1–O6 distances, which were set to (2.0 ± 0.2), (2.9 ± 0.3), (2.0 ± 0.2) and (2.9 ± 0.3) Å respectively.

#### Planarity restraints

Planarity restraints were applied to the G2•G6•G9•G12, G15•G5•G8•G11, G20•G23•G3•G17, and G21•G24•G27•G18 tetrads.

#### Distance-geometry simulated annealing

Initial extended conformation of *AT26* sequence was generated using the XPLOR-NIH (74) program by supplying the available standard topology and parameter tables. Each system was then subjected to distance-geometry simulated annealing by incorporating distance, dihedral, hydrogen bond, and planarity restraints. One hundred structures were generated and subjected to further refinement.

#### Distance-restrained molecular dynamics refinement

The 100 structures obtained from each simulated annealing step were refined with a distance-restrained molecular dynamics protocol incorporating all distance restraints. The system was heated from 300 to 1000 K in 14 ps and allowed to equilibrate for 6 ps, during which force constants for the distance restraints were kept at 2 kcal mol^−1^ Å^−2^. The force constants for non-exchangeable proton and exchangeable proton restraints were then increased to 16 kcal mol^−1^ Å^−2^ and 8 kcal mol^−1^ Å^−2^ respectively in 20 ps before another equilibration at 1000 K for 50 ps. Next, the system was cooled down to 300 K in 42 ps, after which an equilibration was performed for 18 ps. Coordinates of the molecule were saved every 0.5 ps during the last 10.0 ps and averaged. The average structure obtained was then subjected to minimization until the gradient of energy was less than 0.1 kcal mol^−1^. Dihedral (50 kcal mol^−1^ rad^−2^) and planarity (1 kcal mol^−1^ Å^−2^ for tetrads) restraints were maintained throughout the course of refinement. Ten-lowest energy structures were generated.

### Bioinformatics

The basic algorithm for the search of sequences containing eight G_2_ tracts separated by 1–2 non-guanine loops is as follows: ‘[GG+X_1–2_]_7_GG+’, designated as 8G_2_ query, with X represents non guanine bases, i.e. A/C/T. The derivative algorithms for the search of sequences containing seven G_2_ tracts and two isolated guanines separated by 1–2 non-guanine loops (7G_2_+2G_1_ query) is similar to the above algorithm; with the exception of having nine instead of eight ‘G-tracts’, and having ‘G+’ in place of ‘GG+’ in two different places, for a total of 36 different queries (Supplementary Table S2). The two algorithms were matched against the *hg38* database using UNIX grep (Globally search a Regular Expression and Print) command-line utility script. Only the cases of exact matches are reported.

## RESULTS AND DISCUSSION

### NMR and CD spectroscopy of the *AT26* sequence revealed the formation of a four-layered G4 structure

**Figure 1.**
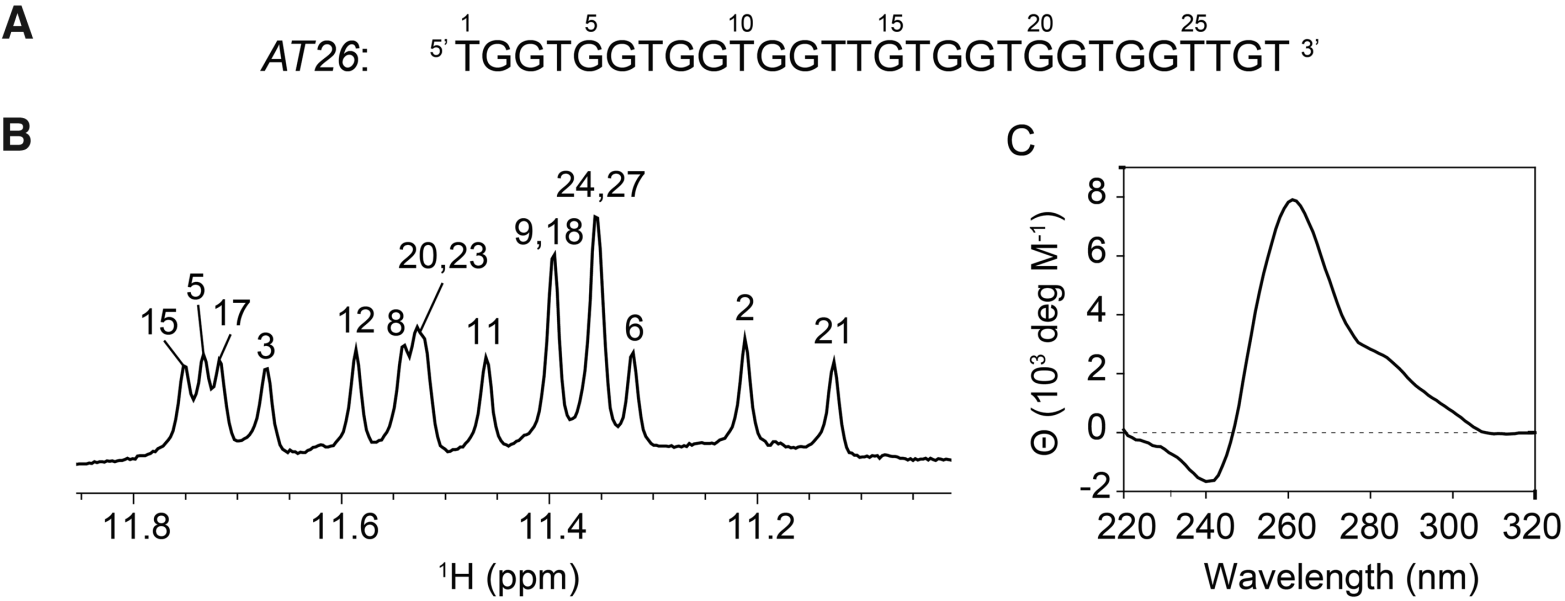
Spectroscopic characterization of *AT26*. (**A**) DNA sequence of *AT26*. (**B**) Imino proton region of the ^1^H NMR spectrum revealed 16 peaks which indicated the formation of a four-layered G-quadruplex. The assignment of each imino proton is shown with the corresponding residue number. (**C**) CD spectrum shows a strong positive peak at 260 nm and a shoulder at ∼290 nm. Samples were prepared in 20 mM KPi buffer (pH 7.0) supplemented with 120 mM KCl.

The *AT26* sequence (Figure 1A) was observed to be folded in the presence of K^+^ ions, and not in Na^+^ or NH4^+^ ions (data not shown). Therefore, further experiments were performed in potassium-containing buffer (120 mM KCl, 20 mM KPi, pH 7.0). The imino proton NMR spectrum of *AT26* displayed 16 peaks of comparable intensity at 11.0–11.8 ppm (Figure 1B), suggesting the formation of a four-layered G4 structure as a single major species in solution. The CD spectrum of *AT26* (Figure 1C) revealed a strong positive peak at 260 nm, characteristic of same-polarity stacking of tetrads in the right-handed parallel-stranded G4 conformation. A shoulder at ∼290 nm was observed, which might either originate from a minor conformation undetectable by NMR or due to the presence of a reverse-polarity stacking of tetrads in the structure (75–77).

### NMR spectral assignment of *AT26*

Site-specific low-enrichment (2%) ^15^N-labeled samples were used for unambiguous assignments of all the imino protons (H1) of *AT26* (Supplementary Figure S1). Several guanine aromatic protons (H8) were unambiguously assigned using 2% ^15^N-labeled samples (Supplementary Figure S2), while the aromatic and methyl protons of 5 out of 12 thymine residues were identified using 4% ^15^N,^13^C-dual labeled samples (Supplementary Figure S3). The rest of the guanine and thymine aromatic and/or methyl protons and sugar protons were assigned according to standard protocols using through-space correlation (^1^H–^1^H NOESY of various mixing times) and through-bond correlation (^1^H–^1^H COSY, ^1^H–^1^H TOCSY, and ^1^H–^13^C-HSQC) NMR experiments. The complete assignments of all sixteen guanine H8 protons and twelve thymine methyl protons are indicated on top of the reference spectra (Supplementary Figures S2 and S3).

### *AT26* forms a four-layered G4 comprised of two bi-layered G4 blocks in opposite polarity

**Figure 2.**
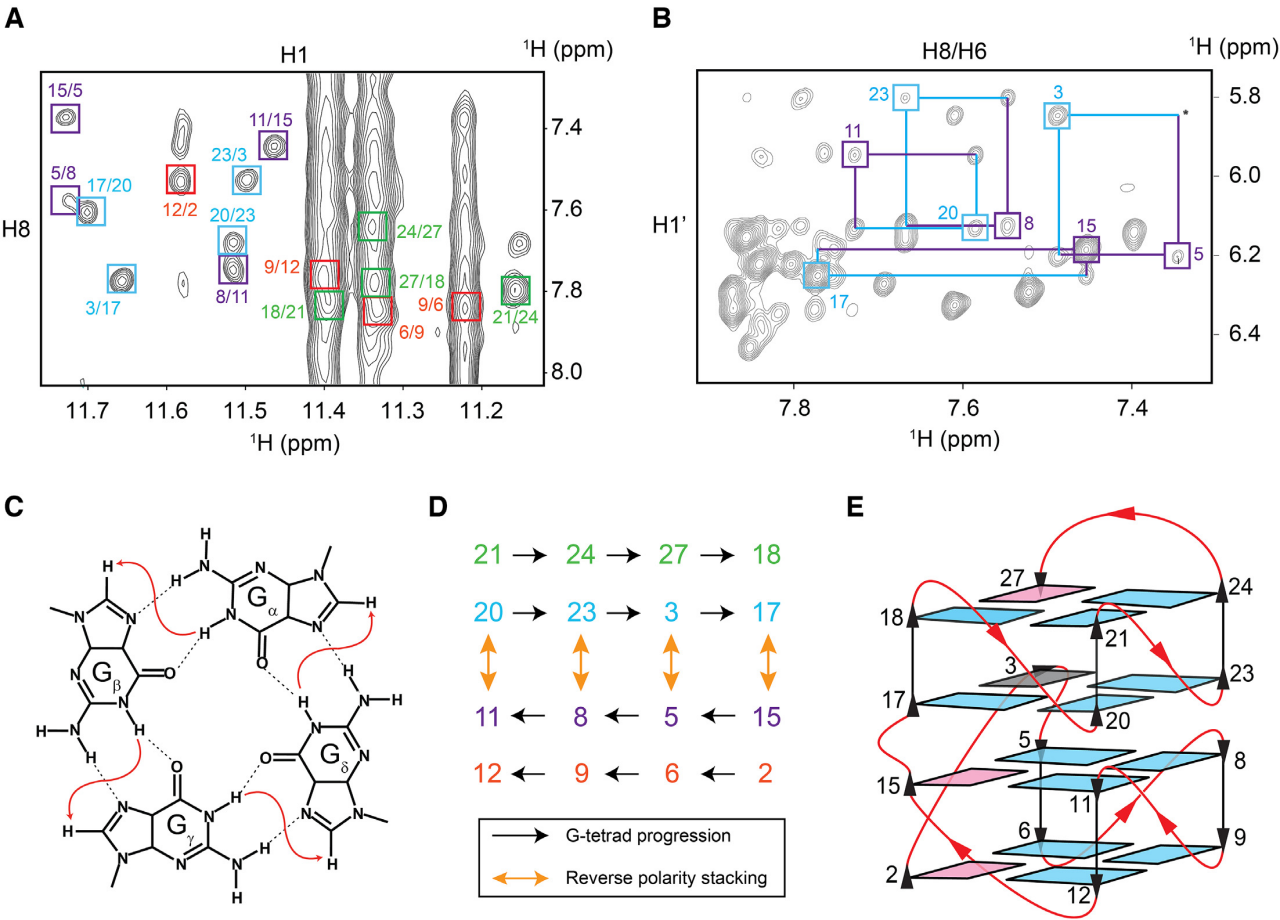
Determination of the folding topology of *AT26*. (**A**) The imino (H1)–H8 region of NOESY spectrum. Characteristic H1–H8 NOE cross-peaks originating from four individual tetrads are boxed and marked in different colors. (**B**) H8–H1′ region of NOESY (mixing time, 300 ms) showing rectangular cross peak patterns for specific guanine pairs from the middle two tetrads, revealing the opposite polarity of the two inner tetrads with respect to each other. (**C**) Schematic of signature cyclic NOE connectivity within a G-tetrad. (**D**) NOE patterns used to establish the four G-tetrads. Each tetrad is represented with a different color. The cyclic H1-H8 connectivity within a tetrad is shown with black arrows and, the reverse polarity stacking between residues of the two inner tetrads are shown with orange arrows. (**E**) Schematic representation of the proposed folding topology of *AT26*. *Anti*- and *syn*-guanine residues are indicated in cyan and magenta respectively. The G3 residue, adopting an undefined glycosidic conformation between *syn* and *anti*, is shown in gray.

The H8–H1′ region of the NOESY spectrum (mixing time, 100 ms) of *AT26* displayed three strong cross-peaks (Supplementary Figure S4), which were identified as the intra-residue H8-H1′ NOE cross-peaks of G2, G15 and G27, indicating *syn* glycosidic bond conformations for these guanines. The remaining 13 guanines exhibited lower intensity intra-residue H8–H1′ cross-peaks, consistent with *anti* glycosidic conformations. The cyclic imino (H1)-H8 NOE patterns, obtained in a NOESY spectrum recorded in H_2_O, identified four individual G-tetrads: G2•G6•G9•G12, G15•G5•G8•G11, G20•G23•G3•G17 and G21•G24•G27•G18 (Figure 2A, C, D). Slow exchange with the solvent was observed for eight out of sixteen guanine imino protons: 1 h after dissolving a dried sample in D_2_O, the eight imino proton peaks of G3, G5, G8, G11, G15, G17, G20 and G23 remained observable, while the other eight imino proton peaks were completely vanished (Supplementary Figure S5). The result implied the solvent-protected position of the specified eight guanine residues, suggesting their localization in the middle two tetrads of the folded four-layered G4 structure. Specific rectangular H8-H1′ NOE cross-peak patterns were observed for guanines of these two middle G-tetrads (Figure 2B). The specific guanine pairs, namely G20↔G11, G3↔G5, G23↔G8 and G17↔G15, revealed the relative positions of guanines in the inner tetrads; together with the knowledge of guanine cyclic connectivity in each tetrad, we deduced that there is a reversal of polarity between the inner two tetrads (Figure 2D). Combining the information from dihedral torsion angle, G-tetrad alignment, solvent exchange analysis and reversal of polarity between the inner tetrads, the folding topology of *AT26* was deduced (Figure 2E): the overall structure of *AT26* is composed of two blocks of bi-layered parallel G4s with opposite polarity; the G-tracts are connected by various loops and bulges. The absence of certain sequential H8-H1′ connectivities in the NOESY spectrum recorded with a mixing time of 300 ms (Supplementary Figure S6) supported the formation of multiple sharp turns in the propeller and V-shaped loops.

### Solution structure of *AT26*

**Figure 3.**
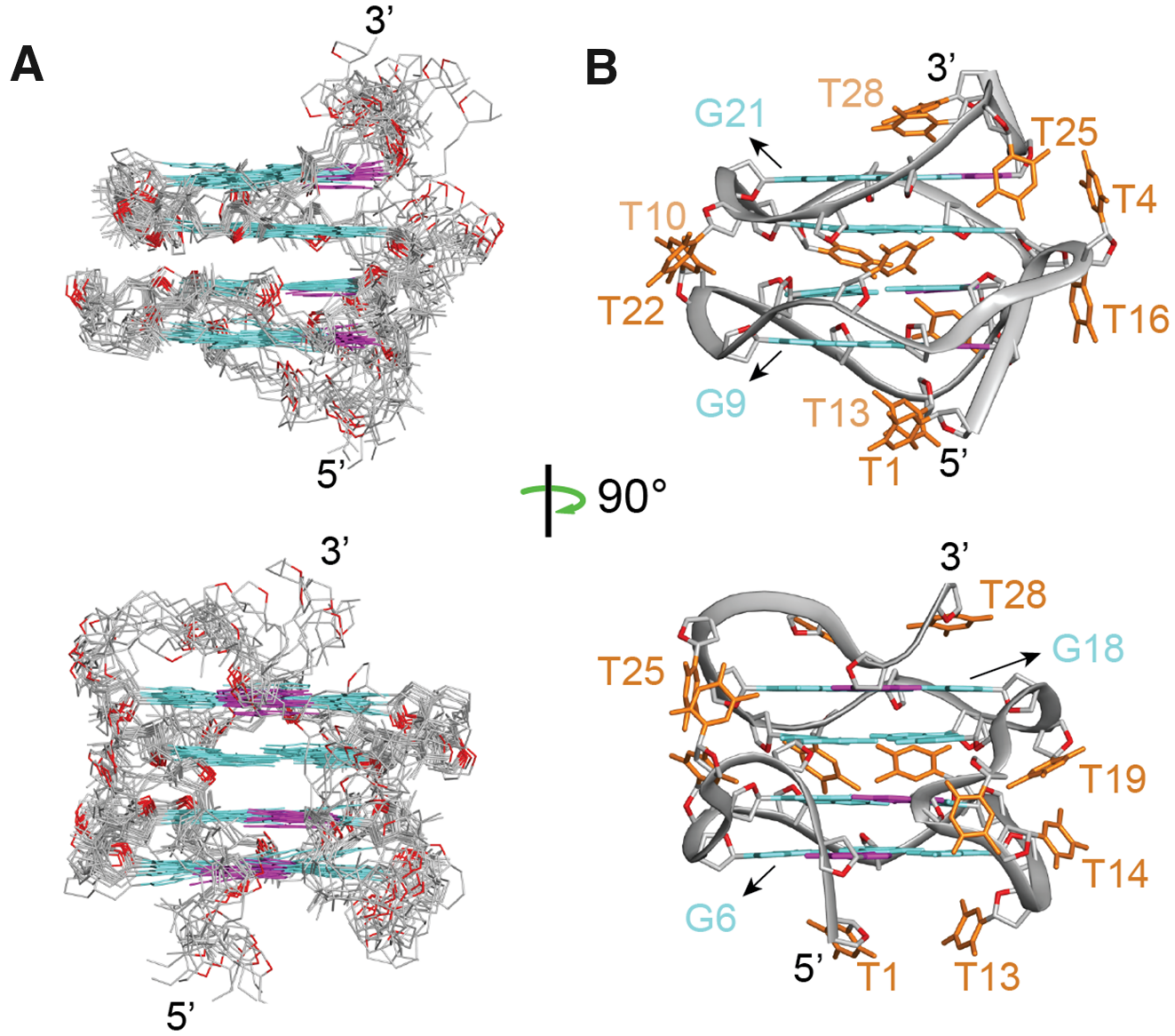
NMR solution structure of *AT26*. (**A**) Superposition of the ten lowest-energy calculated structures of *AT26*. (**B**) Ribbon view of the representative structure of *AT26*. *syn*- and *anti*- guanine bases are indicated in magenta and cyan respectively, except for G3 whose glycosidic conformation falls between *syn* and *anti*; thymine bases are shown in orange; the phosphate backbones are shown in gray; sugar O4′ atoms are shown in red.

The NMR solution structures of *AT26* were calculated based on distance, angle, hydrogen-bond and planarity constraints (Table 1) obtained from the analyses of NMR spectra (see Materials and Methods). The ten lowest-energy structures out of the 100 calculated structures were superimposed and presented (Figure 3A). The ensemble of the ten lowest-energy structures was well converged with a pairwise rmsd value of (0.65 ± 0.16) Å for the G-tetrad core. Ribbon representation of a representative refined structure is presented (Figure 3B).

**Table 1.** NMR restraints and structure statistics

**A. NMR restraints**		
Distance restraints	Exchangeable	Non-exchangeable
Intra-residue	–	422
Inter-residue	51	250
Other restraints		
Hydrogen bond	64	
Dihedral angle	15	
Planarity	4	
**B. Structure statistics**		
NOE violations		
Number (>0.2 Å)	0.000 ± 0.000	
Deviations from the ideal geometry		
Bond lengths (Å)	0.001 ± 0.000	
Bond angles (°)	0.320 ± 0.003	
Impropers (°)	0.191 ± 0.003	
Pairwise heavy atom RMSD value (Å)		
G-tetrad core	0.646 ± 0.160	
All heavy atoms	2.199 ± 0.278	

### Structural elements of *AT26*

#### Sequential and non-sequential G-tracts

**Figure 4.**
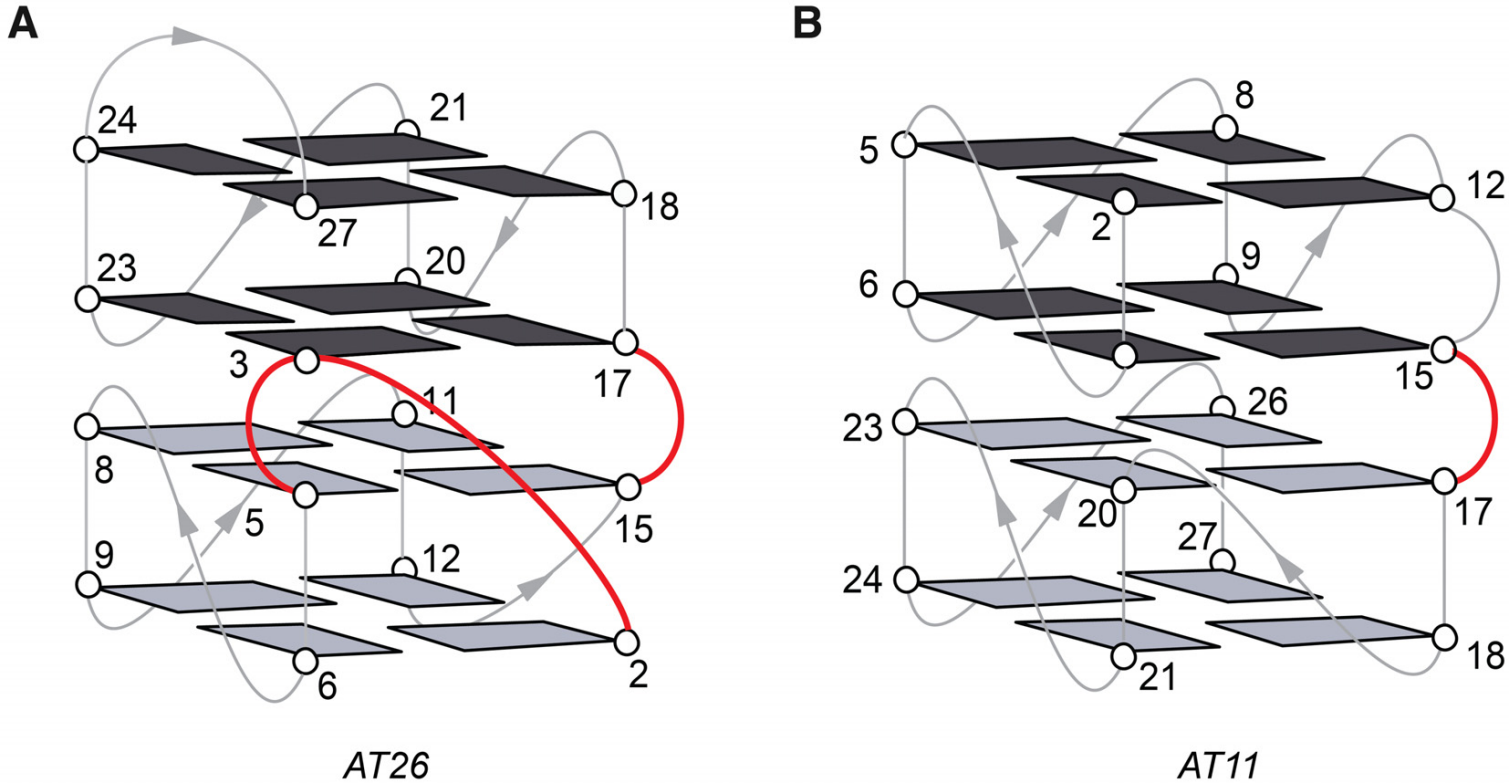
Locking elements in (**A**) *AT26* and (**B**) *AT11*. Guanine bases in two bi-layered blocks are shown in black and gray, respectively; the backbone and loops are shown in gray; the linkers are highlighted in red.

Despite having seven G_2_ tracts in the sequence, the overall folding topology of *AT26* displayed only six G_2_ tracts being part of successive G-tetrads. The first G_2_ tract (G2-G3) spans three tetrad layers, where the residue G2 is involved in the bottom (first) tetrad and the residue G3 is a part of the third tetrad as depicted in the folding schematic (Figures 2E and 4A). The rest of the G_2_ tracts (G5–G6, G8–G9, G11–G12, G17–G18, G20–G21, and G23–G24) are each arranged regularly as two stacking guanines (Figure 2E). The two isolated guanines, G15 and G27, are stacked with G2 and G3 respectively, forming non-sequential ‘G-tracts’. Every pairwise guanine-to-guanine stack is supported by the existence of the corresponding inter-residue cross-peaks in the NOESY spectrum (mixing time, 300 ms) recorded in D_2_O solvent (Supplementary Figure S6).

#### AT26 structure involves three connecting linkers

Linkers are structural elements that connect two or more building blocks and form the basis of the higher-order structural assembly. G4 linkers can be either intramolecular or intermolecular. Intramolecular G4 linkers connect different parts of a sequence which may fold independently. Intramolecular linkers are a common feature in four-layered G4 structures made of short guanine (G_2_ and G_1_) stretches, where they connect tetrads of two bi-layered building blocks. The connection can be between adjacent G-tetrads (Figure 4B and Supplementary Figure S7C) or distant G-tetrads (Supplementary Figure S7D–F). However, in certain cases, bi-layered G4s formed of short G stretches was shown to stack on each other to form stable four-layered G4 without the need for an intramolecular linker; the resulting structures are identical in terms of folding topology except the absence of the linker (Supplementary Figure S7A versus S7D and S7B versus S7E). On the other hand, intermolecular G4 linkers, also known as ‘interlocks’, connect G4 building blocks from two or more strands. Interlocks provide a structural basis to form long and extremely stable G4s from short sequences. For example, the 16-nt *93del* sequence d[GGGGTGGGAGGAGGGT] along with two other derivatives *s2* and *s4*, formed G4s containing six G-tetrads by means of interlocking (Supplementary Figure S7G–I).

The overall structure of *AT26* can be regarded as stacking of two blocks of bi-layered G4s, comparable to those of *AT27* and *AT11* (Figure 4 and Supplementary Figure S7C). However, as opposed to having two independent stacking blocks (78), the two blocks of *AT26* are locked into each other, therefore the name intra-locked G4. There are three bridging points that are responsible for the locking of the two blocks: (i) G2-G3 tract, (ii) the bulge linker T4 and (iii) the other bulge linker T16 (Figure 4A). Furthermore, we showed (see below) that the deletion of both T4 and T16 (*del*T4,16, Table 3) resulted in a significant increase in stability, indicating that tighter locking/interaction between the two bi-layered G4 blocks is favorable. The locking feature signifies the inter-dependency between the folding of the two blocks in *AT26* sequence, in contrast with *AT27* and *AT11* (Figure 4 and Supplementary Figure S7C).

#### Various connecting loops

**Figure 5.**
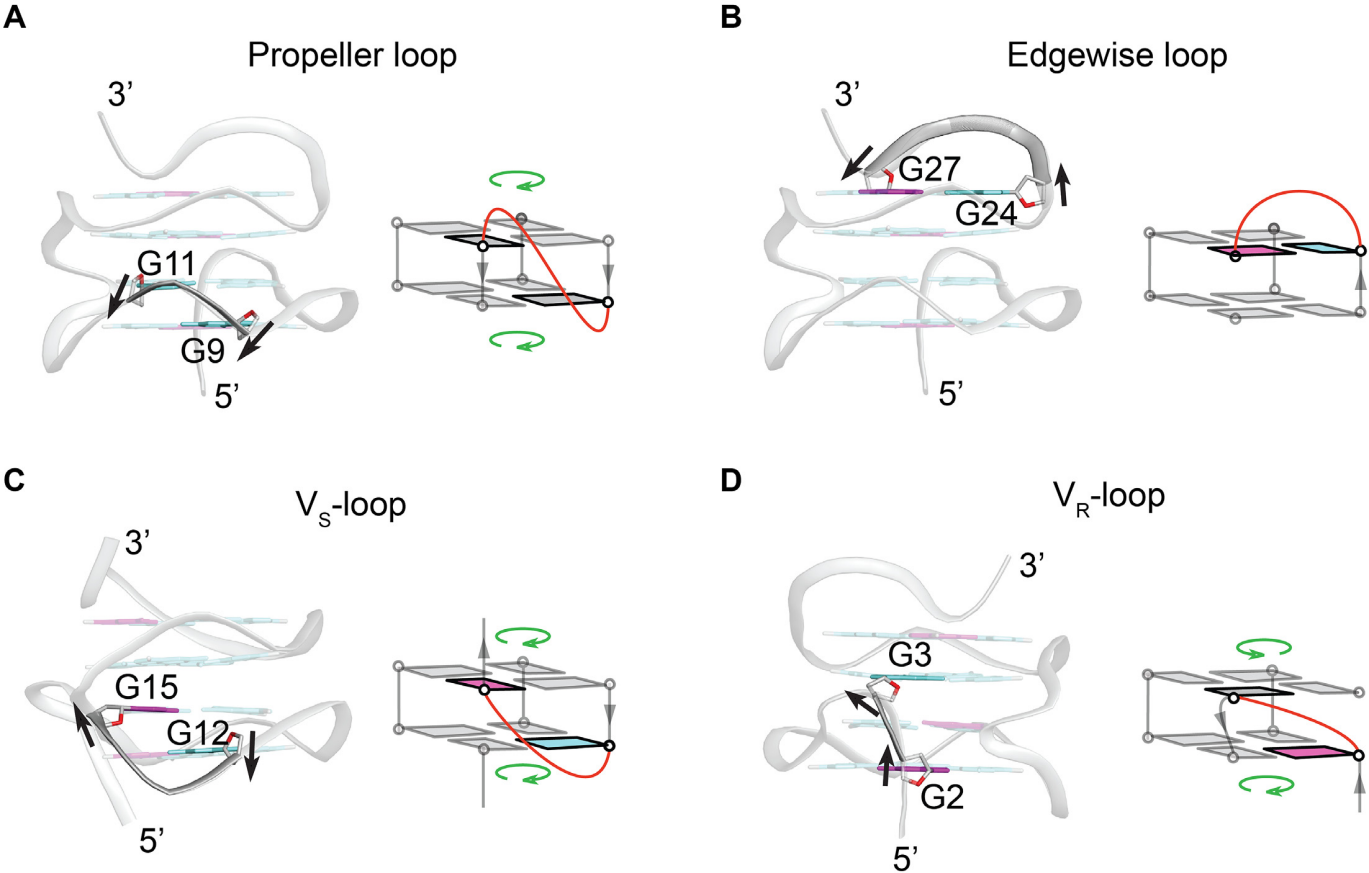
The loops in *AT26*: Each type of loops is shown from the structure together with a general schematic. (**A**) Propeller loop (T10). Propeller loops generally connect two tetrads with same polarity; both guanines involved usually have the same glycosidic conformation. (**B**) Edgewise loop (T25–T26). Edgewise loops connect guanines in the same tetrad, the two guanines usually have different glycosidic conformation. (**C**) V_S_-loop (T13–T14). V_S_-loops may span two or three tetrads, the tetrads have same polarity and one of the two guanine residues being connected by the loop is usually *syn*. (**D**) V_R_-loop (backbone of G2–G3). V_R_-loops may span three or two tetrads; the tetrads have different polarity with respect to each other; and the 5′-dG of the loop is usually *syn*, while the 3′-dG has undefined glycosidic conformation. The arrows roughly indicate the direction of the sugar backbone of the guanines; the loop residues are removed for clarity; the *syn*, *anti*, and undefined guanines are shown in magenta, cyan, and gray respectively, the phosphate backbone is shown in gray, sugar O4′ atoms are shown in red. For the schematic representations, the loops of interest in each panel are highlighted and shown in red and other DNA backbones without any direction information is made transparent and shown in gray; guanine residues other than the ones connected by the loops are shown in gray; the polarity of the tetrads are shown with a green arrow.

There are three types of connecting loops in the *AT26* structure: the propeller loops (T7, T10, T19 and T22), the edgewise loop (T25–T26) and the V-shaped loops (T13-T14 and phosphate backbone between residues G2–G3). Generally, propeller loops are defined as the nucleic acid strands that connect two guanines in different G-tetrad planes pointing to the same direction (Figure 5A), whereas edgewise loops are described as the connecting strands between two guanines from the same G-tetrad plane pointing to different direction (Figure 5B). V-shaped loops connect two different G-tetrads, in which the system has one missing G–G support column (Figure 5C-D) (51). V-shaped loops share some structural features of the previous two loops, yet they are unique in some regards. The relative sugar/backbone orientations of the guanines being connected by the loop may vary, and the participating G-tetrads may have the same or different polarities with respect to each other. In *AT26*, the V-shaped loop T13–T14 connects the G12 and G15 residues situated in first and second tetrads from the bottom respectively. The sugars/backbones of the two involved guanines roughly point in opposite directions (G12 points downward, while G15 points upward), and the two tetrads adopt the same polarity (Figure 5C). The other V-shaped loop, which comprises of the phosphate backbone between G2 and G3, spans three tetrad layers. The sugars/backbones of the two guanines roughly point towards same direction (upward) and there is a reversal of polarity of the two tetrads with respect to each other (Figure 5D). Both of these two types of loops are reported in literature by the name of V-shaped loops. Taking in account the difference in relative polarities of the two tetrads being connected by the V-shaped loops, we propose to classify the loops as V_S_-loop (connects two tetrads with same polarity) (51,79), and V_R_-loop (connects two tetrads with reverse polarity) (45,71,72,80–84) (Supplementary Table S1).

The guanine residue at the 5′-end of the V_R_-loop (5′-dG) of *AT26* (G2) has a *syn* glycosidic conformation. The guanine residue at the 3′-end of the V_R_-loop (3′-dG) of *AT26* (G3) has a glycosidic bond angle of (317.0 ± 6.2)°, which falls in between the range of *syn* and *anti* conformations. A survey of the other reported structures with V_R_-loops consisting of natural DNA nucleotides showed that in all cases, except for 6JCD, the 5′-guanine residue of the V_R_-loop adopts *syn* glycosidic conformation while the 3′-dG residue adopts an *undefined* glycosidic conformation with angle in between *syn* and *anti* (Table 2). In case of 6JCD, the V_R_-loop connects a 5′-dG with *undefined* glycosidic conformation to a 3′-dG adopting *syn* conformation (Table 2) (71). In a G4 structure containing LNA modified nucleotides (PDB code, 2WCN), the V_R_-loops connect both guanine residues with *anti* glycosidic conformation, which could be attributed to the effect of LNA sugar being locked in *anti* conformation (80). A recent report on oligonucleotides containing modified nucleotides showed that beyond the glycosidic angles, north-type sugar puckering of the guanine residues can drive the formation of V-shaped loops (83).

**Table 2.** Summary of glycosidic angle (χ)^a^ of the guanine residues in V_R_-loops consisting of natural DNA nucleotides

PDB code	Measured χ value (°) for 5′-dG	Measured χ value (°) for 3′-dG	Reference
1U64	75.2 ± 6.1 (*syn*)	319.4 ± 0.9 (*undefined*)	(72)
2KPR	40.6 ± 5.6 (*syn*)	313.9 ± 12.7 (*undefined*)	(85)
6H1K	72.2 ± 25.6 (*syn*)	333.3 ± 11.5 (*undefined*)^b^	(45)
5ZEV	77.1 ± 1.2 (*syn*)	275.1 ± 1.1 (*undefined*)	(81)
6A7Y	64.8 ± 5.0 (*syn*)	343.7 ± 6.9 (*undefined*)	(82)
5O4D	87.2 ± 2.4 (*syn*)	274.2 ± 6.8 (*undefined*)	(84)
6KVB	72.3 ± 25.7 (*syn*)	317.0 ± 6.2 (*undefined*)	This work
6JCD	155.4 ± 6.2 (*undefined*)	64.2 ± 3.1 (*syn*)	(71)

^a^The ranges for *anti* and *syn* glycosidic angles (χ) are defined as 240° > χ > 180° and 0 < χ < 90° respectively (86). Glycosidic angles outside of these ranges are marked as *undefined*.

^b^The glycosidic angle extracted from the solution structure (PDB code, 6H1K) falls in the *undefined* range, although the reference described it as a *syn* conformation.

#### Unique groove architectures

The *AT26* structure consists of uniquely assembled grooves (Supplementary Figure S8). There are superpositions of different grooves (wide/medium/narrow) on three out of four sides of the structure. Similar superpositions of different grooves are rare and can be found in very few G4 structures reported so far, such as *93del* (87). Conversely, regular G4 structures with continuous G-tracts [parallel, anti-parallel, or (3+1) hybrid] display uniform grooves on each individual side. Additionally, in the *AT26* structure, different types of loops cover part of the grooves differently (V_S_- and propeller loops spanning two adjacent tetrads and V_R_-loop spanning three tetrads), producing unique available binding surfaces. This distinctive feature is potentially relevant in ligand design for specific targeting of the *AT26* structure.

### Effect of loops and bulges on the structure and stability of *AT26*

Previous studies had indicated that the structural stability of most G4s is inversely related to the lengths of their loops (24–28) and bulges (33). The *AT26* structure, which consists of a 2-nt edgewise loop (T25–T26), a 2-nt V-shaped loop (T13–T14) and two bulges (T4 and T16), has a melting temperature of 40.8°C under ∼150 mM K^+^ condition. The sequence was modified—specifically in the loops and/or bulges—to assess the effects of these structural elements on the structure and stability. The modified sequences, structure formation assessment and thermal stability (Supplementary Figures S9–S11) are listed in Table 3: (i) the augmentation of the edgewise loop from two to three thymines (*ins*T27) retained the structure with increased stability (+3°C), while further increasing the loop to four thymine residues (*ins*T27,28) resulted in decreased stability (–5°C), suggesting the optimum length of the edgewise loop is three residues (Supplementary Figure S11A); (ii) the reduction of the two-thymine V-shaped loop to a one-thymine loop (*del*T13) showed a mixture of at least two G4 conformations, while the augmentation to three (*ins*T15) and four-thymine loop (*ins*T15,16) maintained the structure with decreased stability (–3°C and –7°C respectively), suggesting the optimum length of two residues (Supplementary Figure S11B); (iii) the deletions of the two bulges either individually (*del*T4 and *del*T16) or together (*del*T4,16) resulted in a significant increase in thermal stability, ranging from +10°C to +25°C (Supplementary Figure S11C). The supporting ^1^H NMR and CD spectra of the listed sequences are presented in supplementary materials (Supplementary Figures S9-S11).

**Table 3.** List of modified sequences studied and their thermal stability

Name	Type of study	Sequence (5′-3′)	*T*_m_ (°C)	Δ*T*_m_ (°C)
*AT*26	Original	TGG TGG TGG TGG TTG TGG TGG TGG TTG T	40.8 ± 0.5	-
*ins*T27	Modifications of edgewise loop	TGG TGG TGG TGG TTG TGG TGG TGG TTTG T	44.0 ± 0.5	+3.2
*ins*T27, 28		TGG TGG TGG TGG TTG TGG TGG TGG TTTTG T	35.7 ± 1.2	-5.1
*del*T13	Modifications of V-shaped loop	TGG TGG TGG TGG -TG TGG TGG TGG TTG T	NA^a^	NA^a^
*ins*T15		TGG TGG TGG TGG TTTG TGG TGG TGG TTG T	37.6 ± 0.7	-3.2
*ins*T15,16		TGG TGG TGG TGG TTTTG TGG TGG TGG TTG T	34.0 ± 0.8	-6.8
*del*T4	Deletion of bulges	TGG -GG TGG TGG TTG TGG TGG TGG TTG T	57.0 ± 0.0	+16.2
*del*T16		TGG TGG TGG TGG TTG -GG TGG TGG TTG T	50.6 ± 0.3	+ 9.8
*del*T4,16		TGG -GG TGG TGG TTG -GG TGG TGG TTG T	66.2 ± 0.5	+25.4
*ins*T9	Addition of bulges	TGG TGG TGTG TGG TTG TGG TGG TGG TTG T	NA^a^	NA^a^
*ins*T9,22		TGG TGG TGTG TGG TTG TGG TGTG TGG TTG T	NA^a^	NA^a^
*AT*26*E*	G-tetrad extension	TGG -GG TGG TGG TTG -GG**G** TGG**G** TGG**G** TTG**G**	>90	>+49

^a^NMR spectra indicate the formation of multiple G-quadruplex conformations.

### G4 structure formation from sequences containing irregularly spaced G_2_ tracts and isolated G residues

Most investigated G4 structures contain G-tracts of three or more guanines (G_≥3_) separated by loops, following the most intuitive G4-forming motif (G_3+_N_1–7_G_3+_N_1–7_G_3+_N_1–7_G_3+_) (31). Sequences with short (G_≤2_) and irregularly spaced G-tracts are believed to have relatively lower tendencies to fold into G4 structures since the stability of G4s is directly related to the number of stacking G-tetrad layers.

Examples of G4 structures formed by sequences containing only G_≤2_ tracts include the 15-nt thrombin binding aptamer (*TBA*) sequence (64) and a 19-nt *Bombyx mori* telomeric sequence (66). Both form a two-layered G4 in solution with a non-parallel topology, further stabilized by stacking of structural elements formed by the loop residues. G4 structures with only two G-tetrad layers capped by stabilizing loop elements have also been observed for sequences containing G_≥3_ tracts (88). Sole existence of two-layer parallel G4s without further stacking/stabilizing elements has not been observed. However, two blocks each having two G-tetrad layers could stack on each other to further stabilize the complex, as observed for the (GGA)_8_ sequence consisting of only G_2_ tracts (89). Furthermore, participation of isolated G residues in the G-tetrad core has been documented (33,90–92).

Recently, we reported several examples of G4 structures formed by sequences containing irregularly spaced G_2_ tracts and isolated G residues (Table 4), derived from an anti-proliferative oligonucleotide *AGRO100* (69–71,78,93). Each of these sequences featured seven G_2_ tracts and several isolated guanines connected with single/double thymine residues. The positions of the G_2_ tracts and isolated G residues with respect to each other were varied in these sequences and henceforth the positions of the single/double thymine linkers were changed as well, which resulted in adoption of different structures (Table 4). The thymine residues could form various loops, bulges, or linkers between G4 blocks. Four-layered right-, left-, and mixture right/left hybrid G4s were observed for *AT11*, *AT27* and *TBA-T-Block2* respectively, while *AT21* folds into a two-layered G4 structure accompanied by a knot-like peripheral motif (Figure 4B and Supplementary Figure S7B–C, E–F). The *AT26* structure is yet another example of four-layered G4 formation from a sequence consisting irregularly spaced G_2_ tracts and isolated G residues. The intra-lock motifs in this structure provide tight connections between G4 layers and blocks. Another particular feature is that consecutive guanines in a G_2_ tract (G2–G3) in the sequence do not form adjacent bases in the G-tetrad core. Except for *AT21*, the discussed sequences contained 16 guanines participating in the formation of four G-tetrad layers. For a sequence with more than 16 guanines, such as *AGRO100*, different conformations can be formed and interconverting using different combinations of guanines for the G-tetrad core formation (see below for further discussion on sequence-structure relationship).

**Table 4.** Different G4 folds formed by sequences containing G_2_ tracts and isolated G residues

Name	Sequence (5′-3′)	Type of structure	Handedness	Reference
*AGRO100*	GG TGG TGG TGG TTG TGG TGG TGG TGG	Mixed population	-	( 68 )
*AT11*	TGG TGG TGG TTG TTG TGG TGG TGG TGG T	Four-layered parallel	Right	( 70 )
*AT21*	TGG TGG TGG TGG TTG TGG TTG TGG TGG T	Two-layered anti-parallel with knot-like motif	Right	( 71 )
*2 x Block2*	G TGG TGG TGG TG TT G TGG TGG TGG TGT T	Four-layered parallel	Left	( 78 )
*AT27*	TGG TGG TGG TGG TTG TGG TGG TGG TGT T	Four-layered parallel	Left	( 69 )
*TBA-T-Block2*	GGT TGG TGT GGT TGG TTG TGG TGG TGG TG	Four-layered parallel	Hybrid (left & right-handed)	( 93 )
*AT26*	TGG TGG TGG TGG TTG TGG TGG TGG TTG T	Four-layered parallel	Right	This work

### A small change in sequence can lead to a large change in the G4 fold, but a large change in sequence does not always alter the G4 fold

Despite extensive structural studies on G4-forming sequences over the past three decades, the sequence–structure relationship is yet to be fully understood. Different topologies with different combinations of G-tetrad core and loops have been observed (20–22). We learnt that small changes in sequence or chemical modifications can completely alter the G4 fold (72,94–100). Some rules have emerged to predict the folding topologies and structural elements of G4-forming sequences harboring G_≥3_ tracts, such as the robustness of some loop elements (25,101).

On the other hand, structures of sequences with non-homogeneous G-tract lengths and irregularly spaced G_<3_ tracts have been little explored. The *AGRO100* derivatives (Table 4), consisting of short G_2_ tracts and isolated G residues have provided us an opportunity to understand the effect of small changes in DNA sequences that bring about new folding topologies. As mentioned in the previous section, the structural diversity of these derivatives—which differs very slightly from each other in terms of sequence—suggested that the positions of the isolated guanines and the lengths of the connecting loops are critical determining factors on the adopted folding topologies. To describe it further, consider two highly similar sequences, *AT11* and *AT27*. Both the sequences have the same composition (16 guanines and 12 thymines, Table 4), and they assemble into two bi-layered G4 blocks connected by a linker. However, there are major differences in the backbone progression within these two structures: while *AT11* has a conventional right-handed backbone progression, *AT27* showed a novel left-handed backbone progression. Another intriguing case is the comparison between *AT26* and *AT27*. The *AT26* structure, a right-handed intra-locked scaffold is again dramatically different from that of *AT27*. The difference of the two sequences arises from a base swap at 26th and 27th position, which convert the single thymine loop at position 26 of *AT27* into a double thymine loop at position 25–26 of *AT26*. The role of single thymine loops in favoring left-handed scaffolds was described before (93), providing a possible explanation on completely different structural behavior of *AT26* compared to *AT27*. Yet another *AGRO100* derivative is *AT21* (Table 4), which folds into a two-layered anti-parallel G4 structure, comprising of two edgewise loops and a novel robust knot-like loop motif containing a T•T•G triad and a T•G base pair (71). Note that heavy modifications of the two edgewise loops (nine residues) in *AT21* did not alter the G4 fold. Similarly, multiple sequence mutations can be introduced in the left-handed sequence *2 xBlock2* (78) and *AT26* (this work) without altering the G4 fold.

In summary, a small change in sequence can lead to a large change in the G4 fold, but a large change in sequence does not always alter the G4 fold. The folding landscape of sequences containing irregularly spaced short G-tracts and isolated G residues is complex, and thus it is difficult to formulate sequence-structure relationship rules based on the current understanding. Nevertheless, some observations for the folding of such sequences have emerged, such as (i) a 12-nt minimal left-handed G4 motif GTGGTGGTGGTG that can drive adjacent G-rich sequences in parallel left- or right-handed G4 conformations (78,93); (ii) a 7-nt knot-like motif TGTTGGT that can be formed on top of a G4 structure (71); and (iii) a 11-nt sequence GTGTGGGTGTG that can fold into a stable G-hairpin (102).

### Intra-locked motif as a basis for extension of a G-tetrad core

Possible extension of the *AT26* structure to a five-layered G4 was attempted (*AT26E*), where a guanine residue was added in each of the four elongation positions (marked in boldface, Table 3) of the sequence with both bulges and the 3′-terminal thymine being removed. The formation of G4 structure was confirmed by NMR (Supplementary Figure S9E) and CD experiments (Supplementary Figure S10E). The sequence contains G_2_, G_3_ and G_4_ tracts, presumably forming a five-layered G4 structure (Supplementary Figure S12), which was found to be extremely stable (*T*_m_ > 90°C) under ∼150 mM K^+^ (Supplementary Figure S11D). The data indicate that very stable intra-locked G4s can be formed by sequences with G-tracts of various lengths including several short G_2_ tracts, which might be underestimated previously.

### Prevalence of *AT26*-like sequences in human genome and biological implication

While all *AGRO100*-related sequences listed in Table 4 contain only T and G residues, an earlier study showed that several thymine loop residues could be successfully mutated (individually or concomitantly) to cytosine or adenine residues without altering the fold, broadening the sequence scope for G4 structure formation (78). Here, we performed a bioinformatic search across the whole human genome to find out the prevalence of *AT26*-like motifs, i.e. sequences with short G_2_ tracts and isolated G residues. The general sequence query (G_2+_N_1–2_)_7_G_2+_ (designated as 8G_2_ query), which represents sequences with eight G_≥2_ tracts separated by one/two non-G residues, resulted in 35,216 exact matches (restricting the query even more with exactly G_2_ in each of the eight G-tracts resulted in 12,699 exact matches). Next, we introduced two isolated guanines into the search query, designated as 7G_2_+2G_1_, with seven G_≥2_ tracts and two isolated Gs separated by one/two non-G residues. The total combinations of all possible positions of the isolated Gs produced 36 individual queries (see Materials and Methods and Supplementary Table S2). These 36 query sequences resulted in a total of 109,310 exact matches. Introducing more isolated Gs while decreasing the number of G_≥2_-tracts accordingly (e.g. 6G_2_+4G_1_, 5G_2_+6G_1_, etc.) would much further increase the number of matches. To experimentally test whether it is possible to include more isolated Gs in the sequence while still forming a G4, we introduced one or two more bulges in the *AT26* sequence (Table 3). The resulting sequences which contain six G_2_ tracts with four isolated Gs (*ins*T9), or five G_2_ tracts with six isolated Gs (*ins*T9,22) both forms G4 structure as evident from their NMR (Supplementary Figure S9D) and CD spectra (Supplementary Figure S10D), although we cannot conclude whether the two new sequences fold the same way compared to *AT26*. Together, the bioinformatics and experimental data suggest a high prevalence of *AT26*-like sequences in human genome, which possess G4-forming potential. The abundance of *AT26*-like sequences in human genome may constitute a significant part of the difference between the putative G4 sequences numbers obtained from the ‘Quadparser’ algorithm (∼380,000) (31), the ‘G4Hunter’ algorithm (>2 fold of ∼380,000) (55) and the experimental sequencing technique ‘G4-seq’ (>700,000) (56). As an example, the sequence d(TGGTGGTGGTGGTGGTGGTGGTGGTTGT), which differs from *AT26* by one T-to-G variation (underlined) is found in the human *SYT14* gene, associated with neurodevelopmental abnormalities, spinocerebellar ataxia and glioma cell proliferation (103–105). The presence of a G4 forming sequence in the concerned gene may provide a new therapeutic target for these diseases.

## CONCLUSION

In summary, we have determined the structure of the *AT26* sequence that consists only of G_≤2_ tracts. The resulting structure is a four-layered G4 scaffold. The novel intra-lock mechanism observed in *AT26* distinguishes it from other four-layered G4 structures of related sequences that constitute two independent bi-layered building blocks. In addition, the *AT26* structure features a novel co-existence of two different V-shaped loops in a sequence of natural nucleotides. From the structural study of *AT26* and other variant sequences (*AT11*, *AT27* and *AT21*), it is apparent that G-tract length is not necessarily correlated with number of G-tetrad layers in the folded structure. The position of the isolated G residues in the sequence as well as the loop lengths appear to play critical roles in determining the folding topology of sequences solely containing short G-tracts (G_≤2_). Given the high abundance of *AT26*-like short G_≤2_ tracts containing sequences in the human genome, more structural studies should be devoted to further explore the rules that govern the folding principle of similar sequences.

## DATA AVAILABILITY

The coordinates for the NMR solution structure of *AT26* have been deposited in the Protein Data Bank (PDB ID: 6KVB).

## Supplementary Material

gkaa008_Supplemental_File
