# Supplementary material for: Intra-locked G-quadruplex structures formed by irregular DNA G-rich motifs
Source: Nucleic Acids Res. 2020 Feb 26;48(6):3315–27. doi: 10.1093/nar/gkaa008 (PMC7102960; doi:10.1093/nar/gkaa008)
Supplement: gkaa008_Supplemental_File [file gkaa008_supplemental_file.docx]

_­­­­_**Intra-locked G-quadruplex structures formed by irregular DNA G-rich motifs**

Arijit Maity^1^, Fernaldo Richtia Winnerdy^1^, Weili Denyse Chang^2^, Gang Chen^1,^ and Anh Tuân Phan^1,3,^*

^1^School of Physical and Mathematical Sciences, ^2^School of Biological Sciences and ^3^NTU Institute of Structural Biology, Nanyang Technological University, Singapore

**Supporting Information**


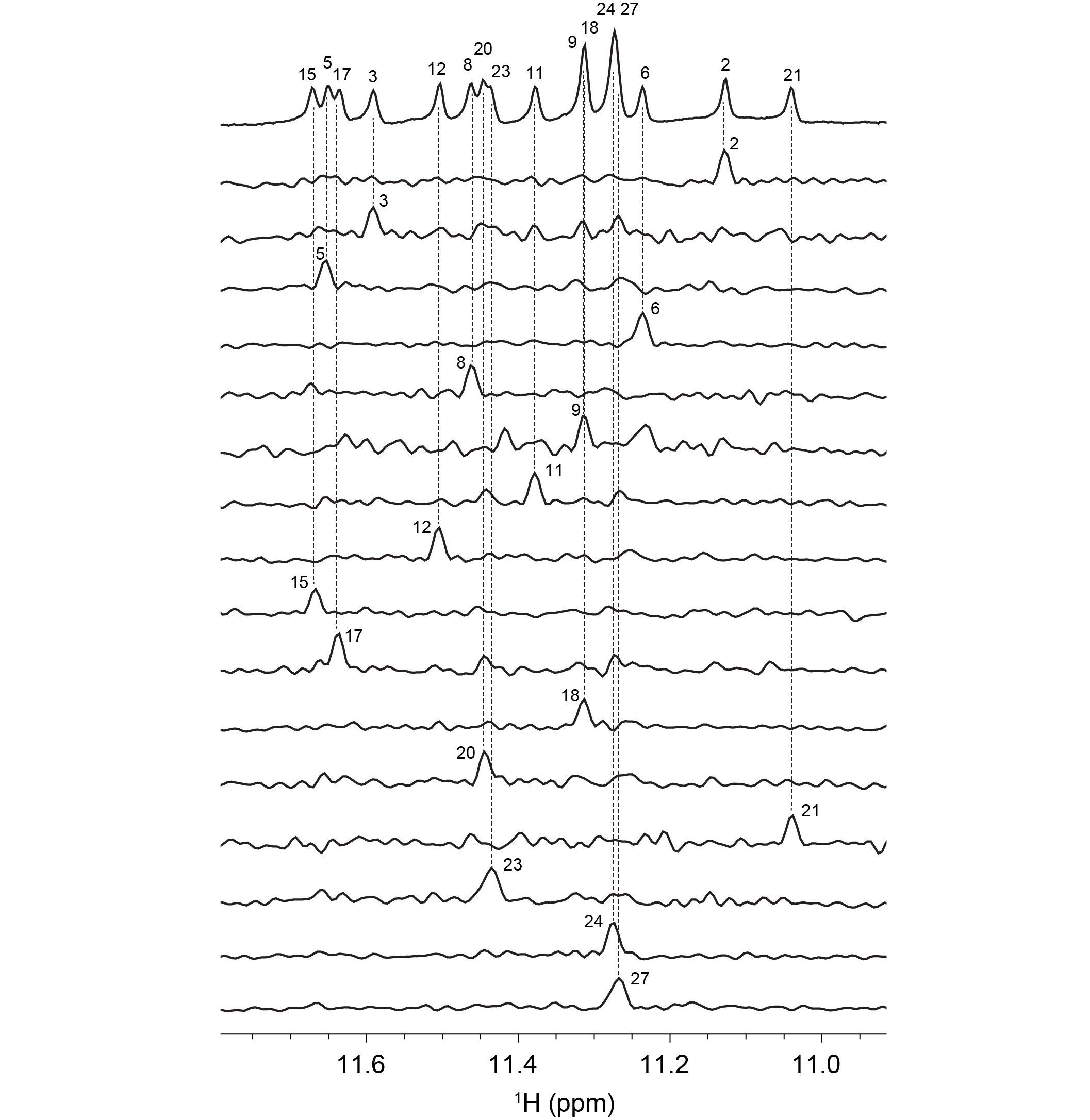


**Fig S1.** Unambiguous assignment of guanine imino (H1) protons by site-specific ^15^N-filtered experiment. The reference spectrum with complete assignment is shown on top.


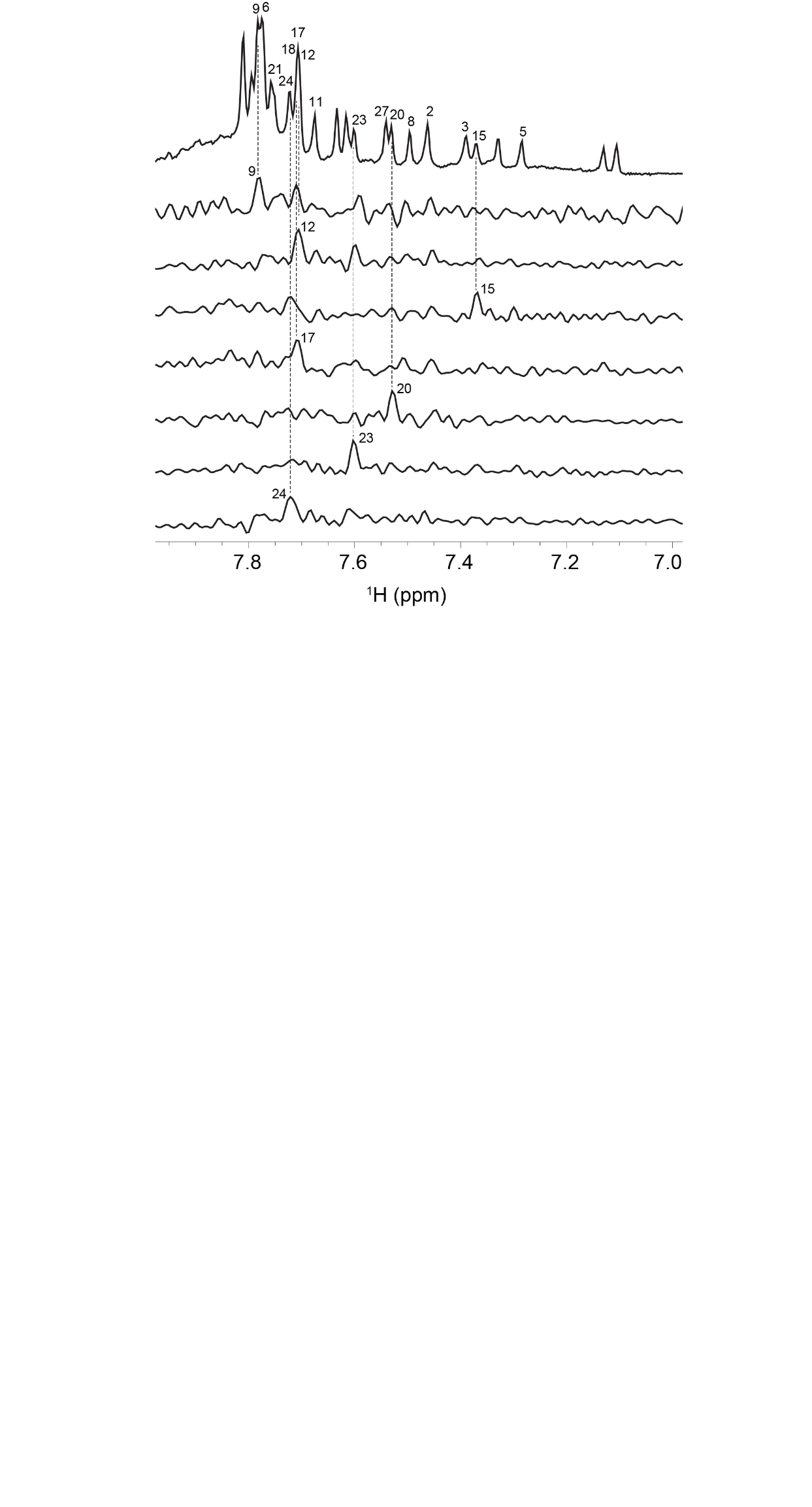


**Fig S2.** Unambiguous assignment of guanine aromatic protons (H8) by site-specific ^15^N-filtered experiment. Seven out of sixteen guanines were assigned using this method, while the rest of the residues were assigned using NOESY, TOCSY and HSQC experiments. The reference spectrum with complete assignment is shown on top.


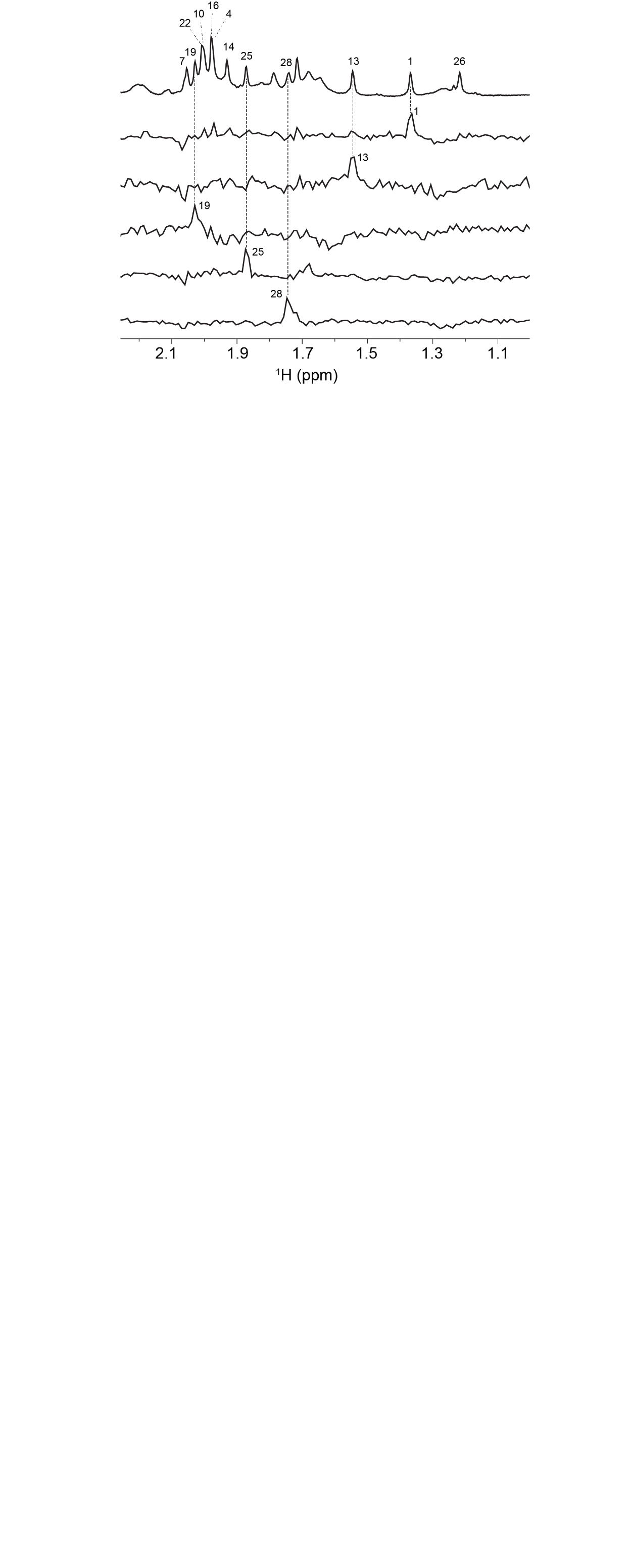


**Fig S3.** Unambiguous assignment of thymine methyl protons by site-specific ^13^C-filtered experiment. Five out of twelve thymine residues were assigned using this method, the rest of the thymine residues were assigned using NOESY, TOCSY and HSQC experiments. The reference spectrum with full assignment is shown on top.


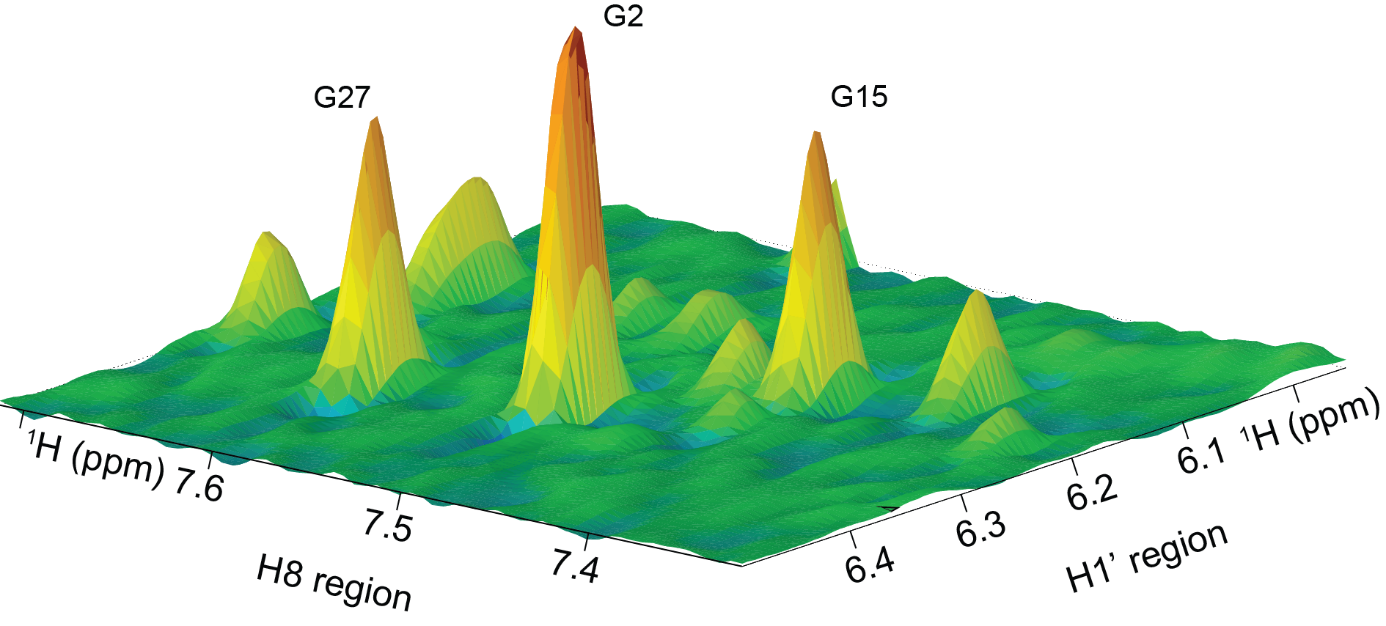


**Fig S4.** Stacked representation of H1’-H8/H6 region of the stacked NOESY plot (100 ms mixing time) recorded in buffer containing 20 mM KPi and 120 mM KCl in 100% D_2_O. The strong cross-peaks for three *syn* residues (labeled on figure) can be distinguished from those of *anti* residues.


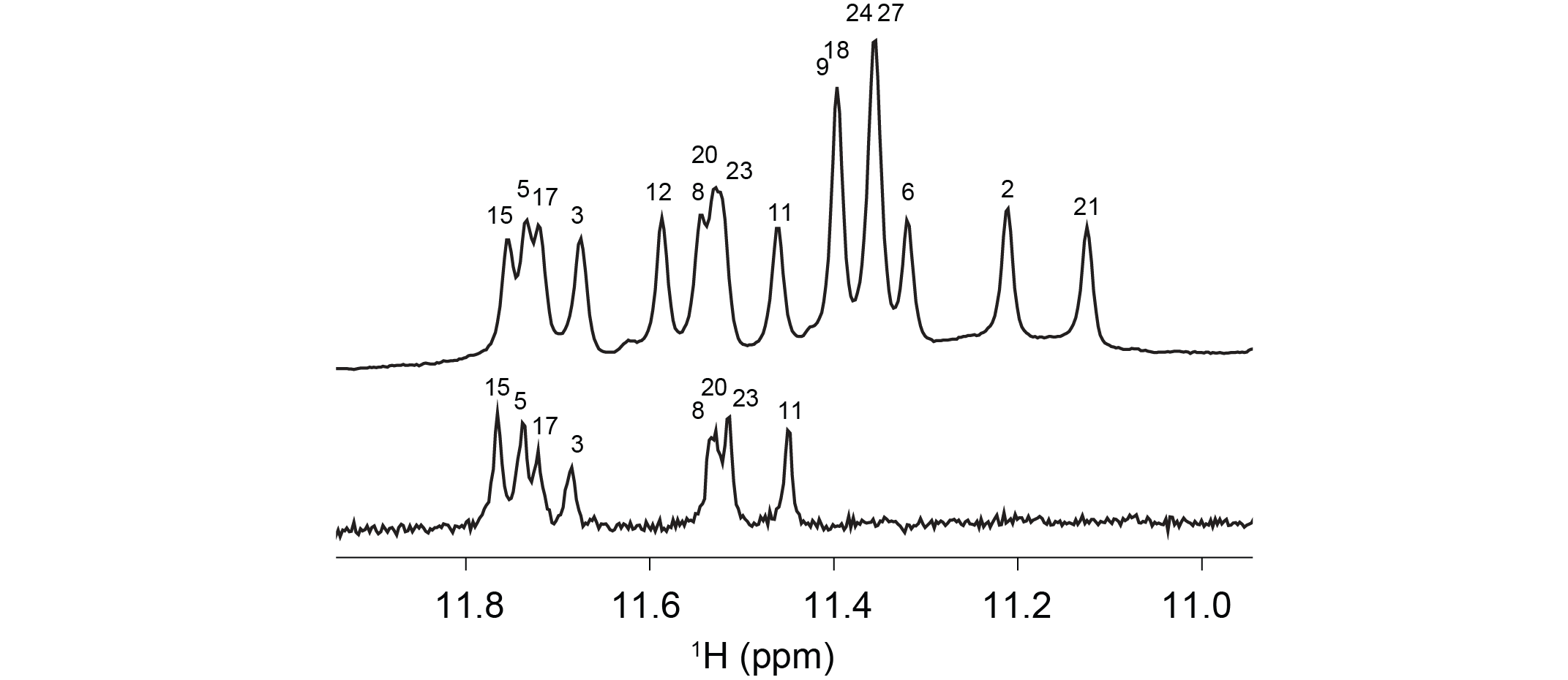


**Fig S5.** Solvent exchange analysis of AT26 at 25 °C. The top spectrum was recorded in 90% H_2_O and 10% D_2_O containing buffer. The bottom spectrum was recorded one hour after adding 100% D2O to the sample.


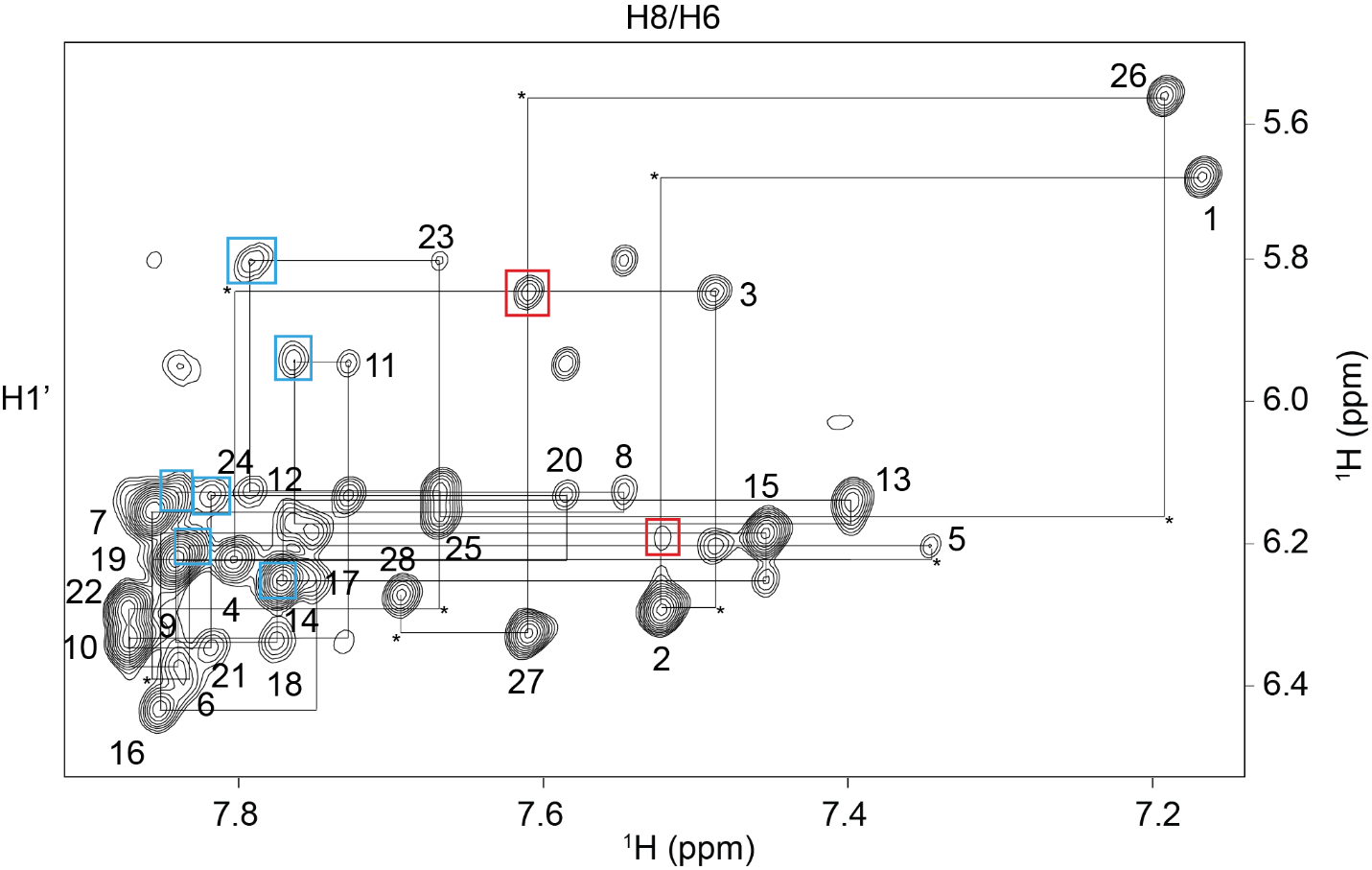


**Fig S6.** H1’-H8 region of NOESY spectrum of mixing time 300ms showing sequential H8_(n)_ – H1’_(n_) – H8_(n+1)_ backbone connectivity. Corresponding residue numbers are marled for intra-residue H8_(n)_ – H1’_(n_) cross peaks. Weak or missing inter-residue cross peaks are represented with *. The sequential guanine cross-peaks for six G_2_ tracts (G5•G6, G8•G9, G11•G12, G17•G18, G20•G21, G22•G23) are shown inside cyan boxes and, the split G cross-peaks (G2•G15 and G3•G27) in red boxes. The experiment was done in 20mM KPi buffer of pH 7.0 supplemented with 120 mM KCl.


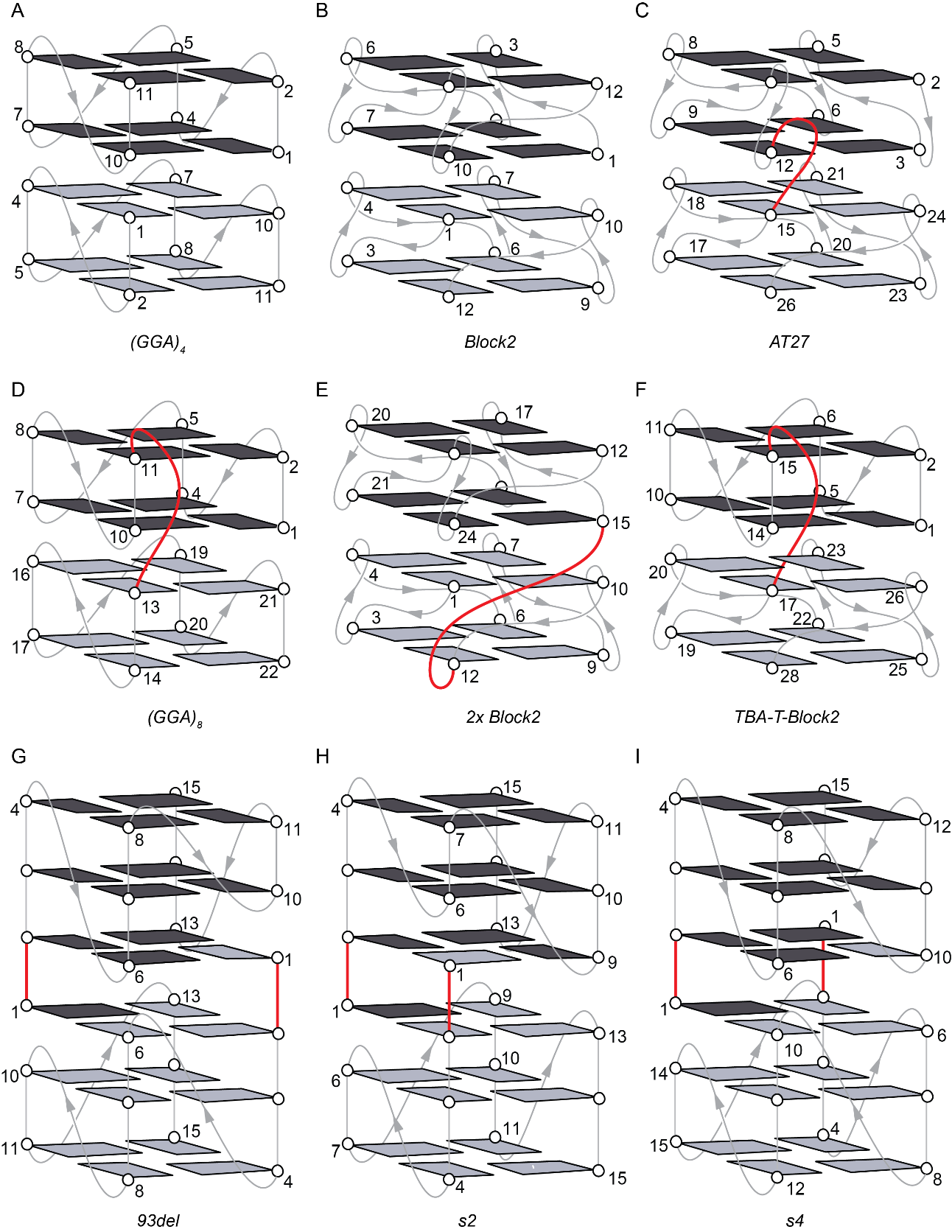


**Fig S7.** Schematics of structures with different type of linkers. (A-B) Bi-molecular G4 structures where two independently folded bi-layered G4 blocks stack upon each without any linker; (C-F) G4 structures where two bi-molecular blocks are connected with a single linker. The linker may connect adjacent (AT27) or distant G-tetrads ((GGA)_8_ and 2x block2). (G-I) G4 structures with intermolecular linker or ‘interlock’. In each schematic guanine bases in two different building blocks are shown in black and gray; the backbone and loops are shown in gray and, the linkers are shown in red. The schematics of structures used in this figure are as follows A) (GGA)_4_ (1), B) Block2 (2), C) AT27(3), D) (GGA)_8_ (4), E) 2x block2 (2), F) TBA-T-block2 (5), G) 93del (6), H) s2 (7) and, I) s4 (7).


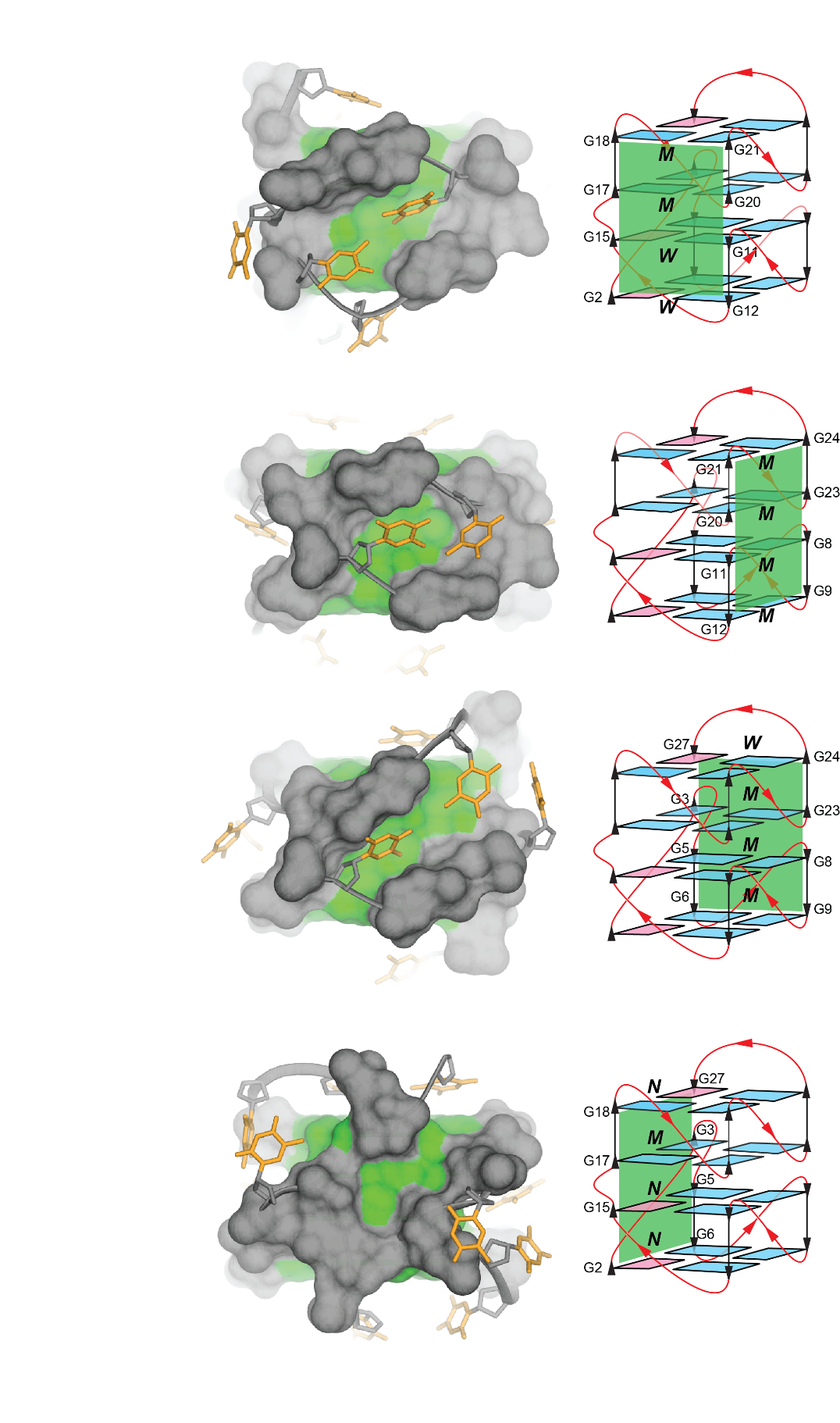


**Fig S8.** Van der Waals surface representation of the AT26 structure from four sides. On the surface representations (left), guanine bases are colored in green; sugar and DNA backbone is shown in gray; and thymine bases are show in orange. On the schematics (right), the side of interest in shown with a green plane next to it. Groove size for each tetrad on the side of interest is shown with letters N, M, and W representing narrow, medium, and wide grooves, respectively. Syn and Anti bases are colored in magenta and cyan.


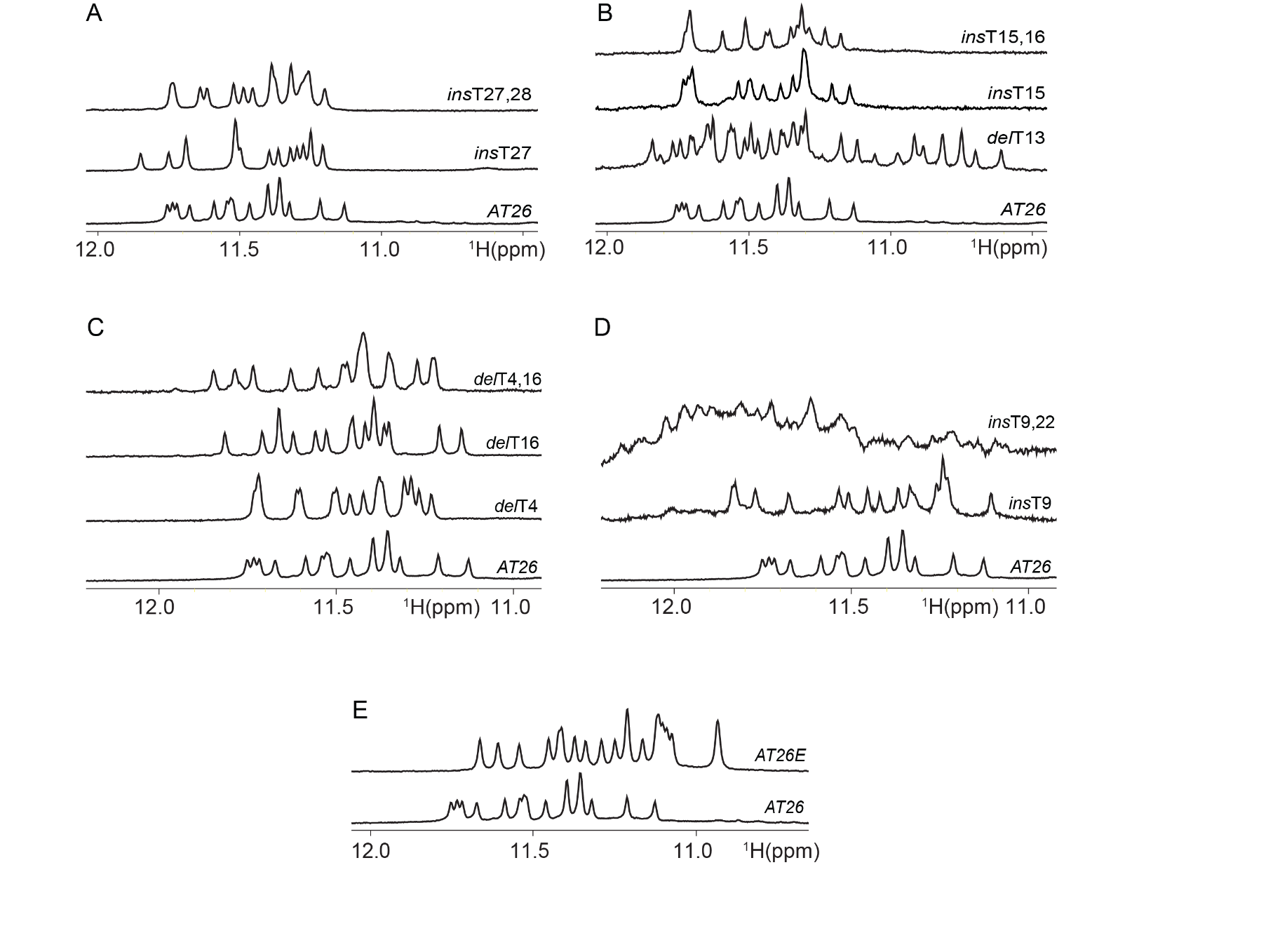


**Fig S9.** NMR spectra of modified sequences. (A) edgewise loop modification, (B) V-shaped loop modifications, (C) deletion of bulges, (D) addition of bulges and, (E) G-tetrad elongation.


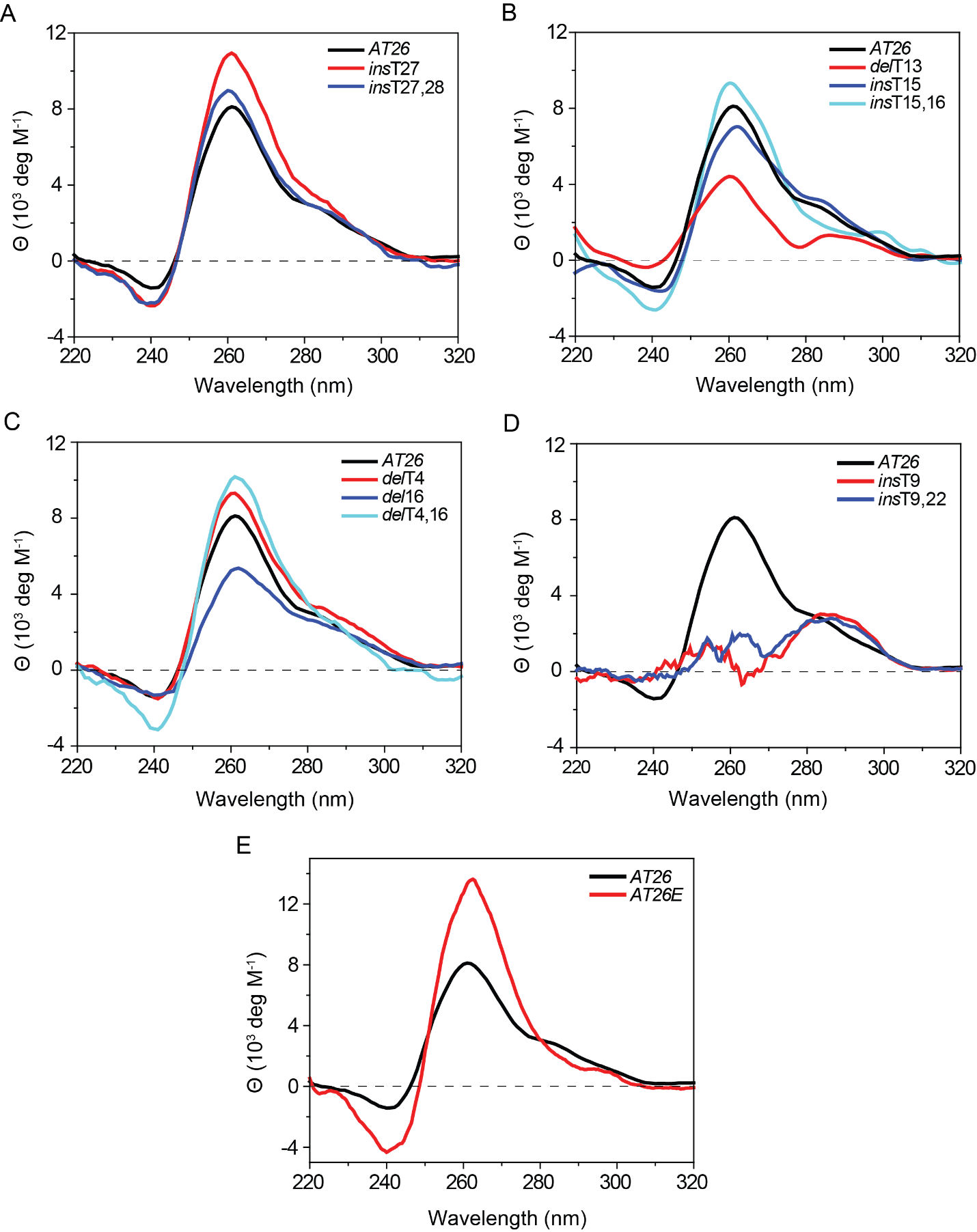


**Fig S10.** CD spectra of modified sequences. (A) edgewise loop modification, (B) V-shaped loop modifications, (C) deletion of bulges, (D) addition of bulges and, (E) G-tetrad elongation.


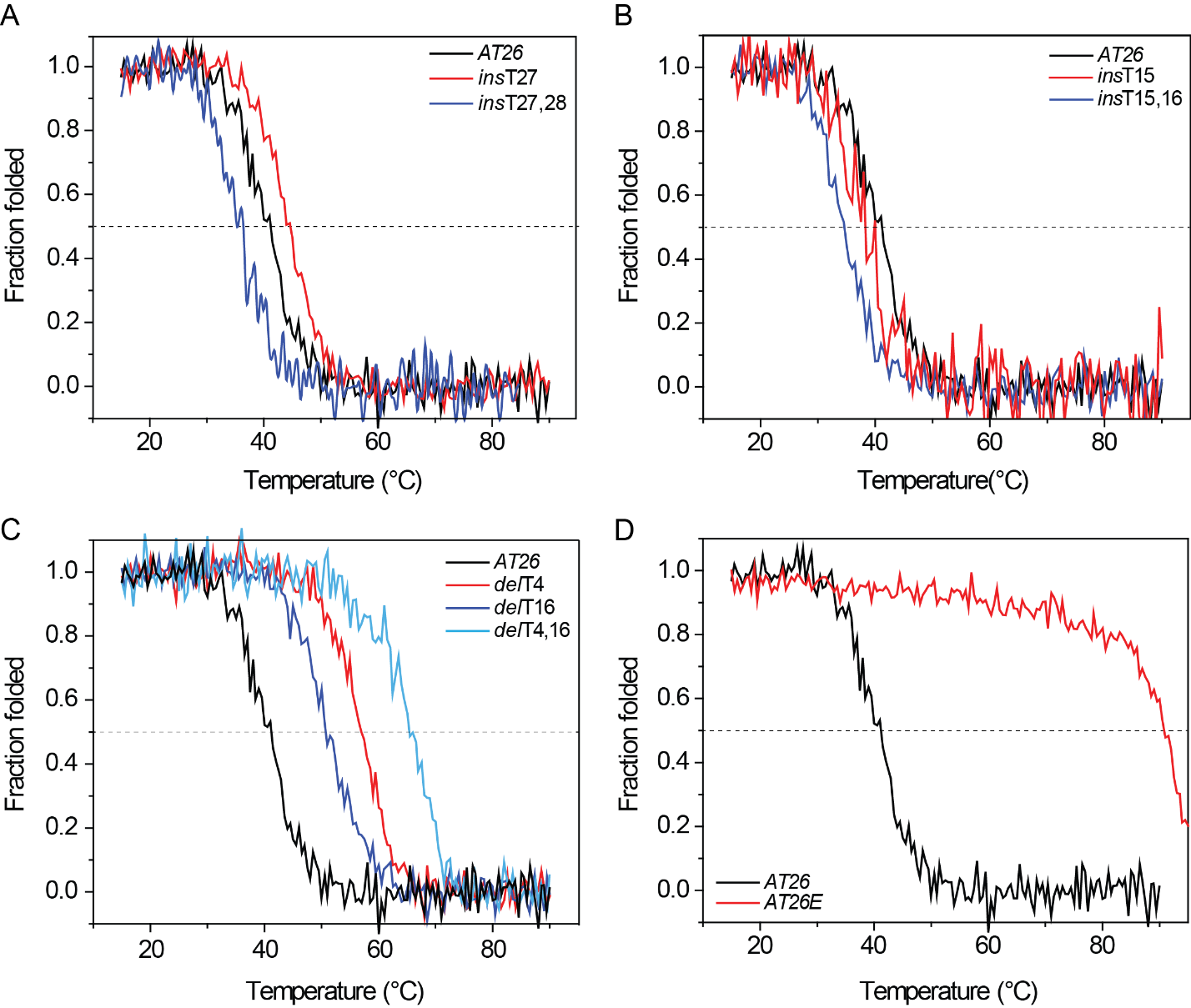


**Fig S11.** CD melting profiles for the comparison of thermal stabilities of *AT26* with sequences consisting of (A) edgewise loop modification, (B) V-shaped loop modifications, (C) deletion of bulges, (D) G-tetrad elongation. Samples were prepared in 20mM KPi buffer supplemented with 12 mM KCl at pH7.0, sample concentration for all thermal melting experiments was 5 µM. Data shown here belongs to the unfolding event only i.e. while increasing the temperature from 15 to 90 °C (95 °C for *AT26E*).


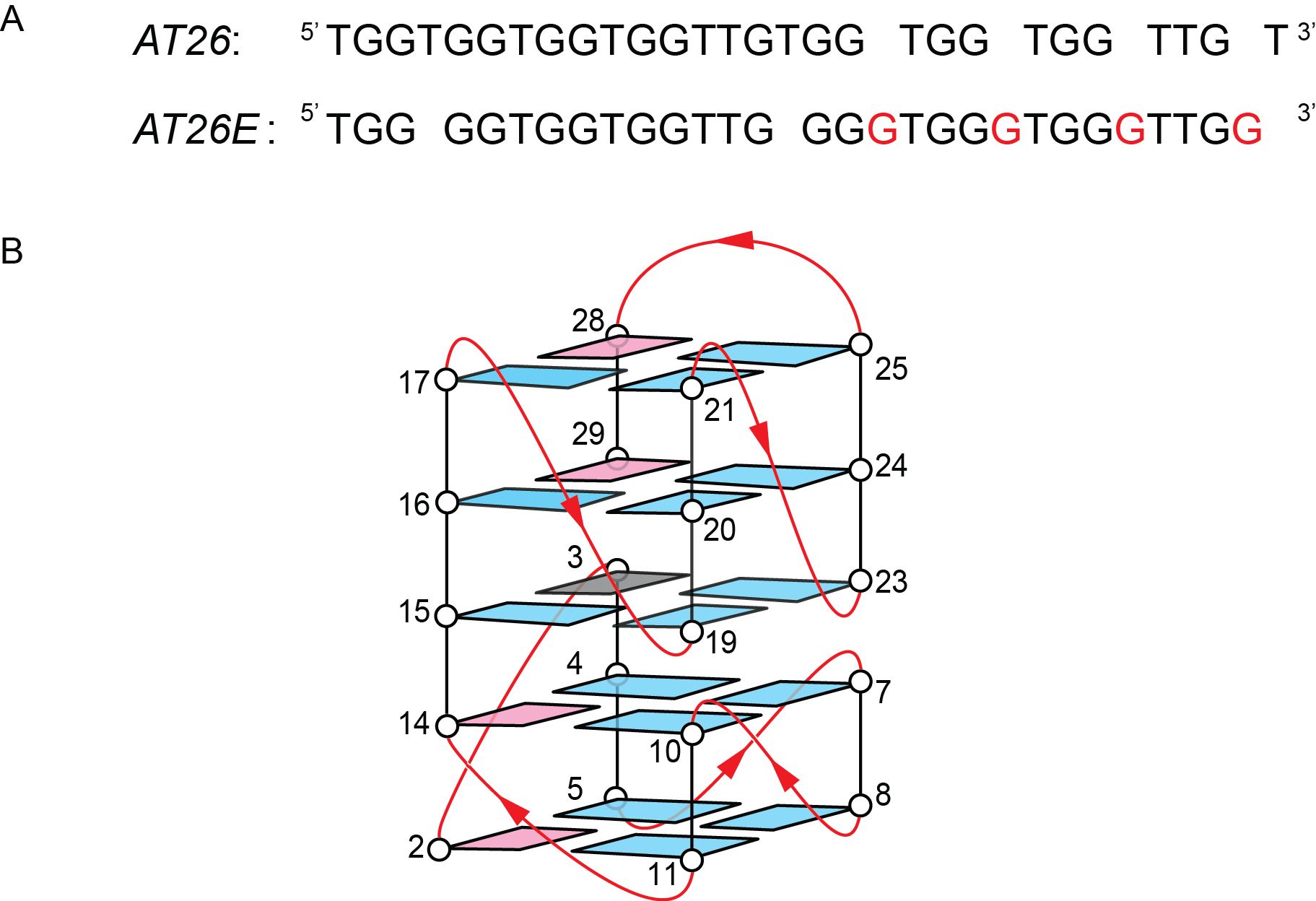


**Fig S12.** Comparison of sequence with AT26 (A), and Schematic (B) of the extended five-layered G4 structure (AT26E). Anti- and syn-guanine residues are indicated in cyan and magenta respectively.

**Table S1. V-shaped loops**

| **Loop type** | **Polarities of connected G-tetrads** | **References** |
| --- | --- | --- |
| V_S_ | Same | (8,9) |
| V_R_ | Reversed | (10-18) |

**Table S2. Bioinformatic search queries for 7G_2_+2G_1_ sequences**

| **No.** | **Sequence** |
| --- | --- |
| 1. | GG+X_1-2_GG+X_1-2_ GG+X_1-2_GG+X_1-2_GG+X_1-2_GG+X_1-2_GG+X_1-2_**G+**X_1-2_**G+** |
| 2. | GG+X_1-2_GG+X_1-2_ GG+X_1-2_GG+X_1-2_GG+X_1-2_GG+X_1-2_**G+**X_1-2_GG+X_1-2_**G+** |
| 3. | GG+X_1-2_GG+X_1-2_ GG+X_1-2_GG+X_1-2_GG+X_1-2_**G+**X_1-2_GG+X_1-2_GG+X_1-2_**G+** |
| 4. | GG+X_1-2_GG+X_1-2_ GG+X_1-2_GG+X_1-2_**G+**X_1-2_GG+X_1-2_GG+X_1-2_GG+X_1-2_**G+** |
| 5. | GG+X_1-2_GG+X_1-2_ GG+X_1-2_**G+**X_1-2_GG+X_1-2_GG+X_1-2_GG+X_1-2_GG+X_1-2_**G+** |
| 6. | GG+X_1-2_GG+X_1-2_ **G+**X_1-2_GG+X_1-2_GG+X_1-2_GG+X_1-2_GG+X_1-2_GG+X_1-2_**G+** |
| 7. | GG+X_1-2_**G+**X_1-2_ GG+X_1-2_GG+X_1-2_GG+X_1-2_GG+X_1-2_GG+X_1-2_GG+X_1-2_**G+** |
| 8. | **G+**X_1-2_GG+X_1-2_ GG+X_1-2_GG+X_1-2_GG+X_1-2_GG+X_1-2_GG+X_1-2_GG+X_1-2_**G+** |
| 9. | GG+X_1-2_GG+X_1-2_ GG+X_1-2_GG+X_1-2_GG+X_1-2_GG+X_1-2_**G+**X_1-2_**G+**X_1-2_GG+ |
| 10. | GG+X_1-2_GG+X_1-2_ GG+X_1-2_GG+X_1-2_GG+X_1-2_**G+**X_1-2_GG+X_1-2_**G+**X_1-2_GG+ |
| 11. | GG+X_1-2_GG+X_1-2_ GG+X_1-2_GG+X_1-2_**G+**X_1-2_GG+X_1-2_GG+X_1-2_**G+**X_1-2_GG+ |
| 12. | GG+X_1-2_GG+X_1-2_ GG+X_1-2_**G+**X_1-2_GG+X_1-2_GG+X_1-2_GG+X_1-2_**G+**X_1-2_GG+ |
| 13. | GG+X_1-2_GG+X_1-2_ **G+**X_1-2_GG+X_1-2_GG+X_1-2_GG+X_1-2_GG+X_1-2_**G+**X_1-2_GG+ |
| 14. | GG+X_1-2_**G+**X_1-2_ GG+X_1-2_GG+X_1-2_GG+X_1-2_GG+X_1-2_GG+X_1-2_**G+**X_1-2_GG+ |
| 15. | **G+**X_1-2_GG+X_1-2_ GG+X_1-2_GG+X_1-2_GG+X_1-2_GG+X_1-2_GG+X_1-2_**G+**X_1-2_GG+ |
| 16. | GG+X_1-2_GG+X_1-2_GG+X_1-2_GG+X_1-2_GG+X_1-2_**G+**X_1-2_**G+**X_1-2_GG+X_1-2_GG+ |
| 17. | GG+X_1-2_GG+X_1-2_GG+X_1-2_GG+X_1-2_**G+**X_1-2_GG+X_1-2_**G+**X_1-2_GG+X_1-2_GG+ |
| 18. | GG+X_1-2_GG+X_1-2_GG+X_1-2_**G+**X_1-2_GG+X_1-2_GG+X_1-2_**G+**X_1-2_GG+X_1-2_GG+ |
| 19. | GG+X_1-2_GG+X_1-2_**G+**X_1-2_GG+X_1-2_GG+X_1-2_GG+X_1-2_**G+**X_1-2_GG+X_1-2_GG+ |
| 20. | GG+X_1-2_**G+**X_1-2_GG+X_1-2_GG+X_1-2_GG+X_1-2_GG+X_1-2_**G+**X_1-2_GG+X_1-2_GG+ |
| 21. | **G+**X_1-2_GG+X_1-2_GG+X_1-2_GG+X_1-2_GG+X_1-2_GG+X_1-2_**G+**X_1-2_GG+X_1-2_GG+ |
| 22. | GG+X_1-2_GG+X_1-2_GG+X_1-2_GG+X_1-2_**G+**X_1-2_**G+**X_1-2_GG+X_1-2_GG+X_1-2_GG+ |
| 23. | GG+X_1-2_GG+X_1-2_GG+X_1-2_**G+**X_1-2_GG+X_1-2_**G+**X_1-2_GG+X_1-2_GG+X_1-2_GG+ |
| 24. | GG+X_1-2_GG+X_1-2_**G+**X_1-2_GG+X_1-2_GG+X_1-2_**G+**X_1-2_GG+X_1-2_GG+X_1-2_GG+ |
| 25. | GG+X_1-2_**G+**X_1-2_GG+X_1-2_GG+X_1-2_GG+X_1-2_**G+**X_1-2_GG+X_1-2_GG+X_1-2_GG+ |
| 26. | **G+**X_1-2_GG+X_1-2_GG+X_1-2_GG+X_1-2_GG+X_1-2_**G+**X_1-2_GG+X_1-2_GG+X_1-2_GG+ |
| 27. | GG+X_1-2_GG+X_1-2_GG+X_1-2_**G+**X_1-2_**G+**X_1-2_GG+X_1-2_GG+X_1-2_GG+X_1-2_GG+ |
| 28. | GG+X_1-2_GG+X_1-2_**G+**X_1-2_GG+X_1-2_**G+**X_1-2_GG+X_1-2_GG+X_1-2_GG+X_1-2_GG+ |
| 29. | GG+X_1-2_**G+**X_1-2_GG+X_1-2_GG+X_1-2_**G+**X_1-2_GG+X_1-2_GG+X_1-2_GG+X_1-2_GG+ |
| 30. | **G+**X_1-2_GG+X_1-2_GG+X_1-2_GG+X_1-2_**G+**X_1-2_GG+X_1-2_GG+X_1-2_GG+X_1-2_GG+ |
| 31. | GG+X_1-2_GG+X_1-2_**G+**X_1-2_**G+**X_1-2_GG+X_1-2_GG+X_1-2_GG+X_1-2_GG+X_1-2_GG+ |
| 32. | GG+X_1-2_**G+**X_1-2_GG+X_1-2_**G+**X_1-2_GG+X_1-2_GG+X_1-2_GG+X_1-2_GG+X_1-2_GG+ |
| 33. | **G+**X_1-2_GG+X_1-2_GG+X_1-2_**G+**X_1-2_GG+X_1-2_GG+X_1-2_GG+X_1-2_GG+X_1-2_GG+ |
| 34. | GG+X_1-2_**G+**X_1-2_**G+**X_1-2_GG+X_1-2_GG+X_1-2_GG+X_1-2_GG+X_1-2_GG+X_1-2_GG+ |
| 35. | **G+**X_1-2_GG+X_1-2_**G+**X_1-2_GG+X_1-2_GG+X_1-2_GG+X_1-2_GG+X_1-2_GG+X_1-2_GG+ |
| 36. | **G+**X_1-2_**G+**X_1-2_GG+X_1-2_GG+X_1-2_GG+X_1-2_GG+X_1-2_GG+X_1-2_GG+X_1-2_GG+ |

**References**

1. Matsugami, A., Ouhashi, K., Kanagawa, M., Liu, H., Kanagawa, S., Uesugi, S. and Katahira, M. (2001) An intramolecular quadruplex of (GGA)(4) triplet repeat DNA with a G:G:G:G tetrad and a G(:A):G(:A):G(:A):G heptad, and its dimeric interaction. *J Mol Biol*, **313**, 255-269.

2. Bakalar, B., Heddi, B., Schmitt, E., Mechulam, Y. and Phan, A.T. (2019) A Minimal Sequence for Left-Handed G-Quadruplex Formation. *Angew Chem Int Ed Engl*, **58**, 2331-2335.

3. Chung, W.J., Heddi, B., Schmitt, E., Lim, K.W., Mechulam, Y. and Phan, A.T. (2015) Structure of a left-handed DNA G-quadruplex. *Proc Natl Acad Sci U S A*, **112**, 2729-2733.

4. Matsugami, A., Okuizumi, T., Uesugi, S. and Katahira, M. (2003) Intramolecular higher order packing of parallel quadruplexes comprising a G:G:G:G tetrad and a G(:A):G(:A):G(:A):G heptad of GGA triplet repeat DNA. *J Biol Chem*, **278**, 28147-28153.

5. Winnerdy, F.R., Bakalar, B., Maity, A., Vandana, J.J., Mechulam, Y., Schmitt, E. and Phan, A.T. (2019) NMR solution and X-ray crystal structures of a DNA molecule containing both right- and left-handed parallel-stranded G-quadruplexes. *Nucleic Acids Res*, **47**, 8272-8281.

6. Phan, A.T., Kuryavyi, V., Ma, J.B., Faure, A., Andreola, M.L. and Patel, D.J. (2005) An interlocked dimeric parallel-stranded DNA quadruplex: a potent inhibitor of HIV-1 integrase. *Proc Natl Acad Sci U S A*, **102**, 634-639.

7. Phan, A.T. and Do, N.Q. (2013) Engineering of interlocked DNA G-quadruplexes as a robust scaffold. *Nucleic Acids Res*, **41**, 2683-2688.

8. Zhang, N., Gorin, A., Majumdar, A., Kettani, A., Chernichenko, N., Skripkin, E. and Patel, D.J. (2001) V-shaped scaffold: a new architectural motif identified in an A x (G x G x G x G) pentad-containing dimeric DNA quadruplex involving stacked G(anti) x G(anti) x G(anti) x G(syn) tetrads. *J Mol Biol*, **311**, 1063-1079.

9. Adrian, M., Ang, D.J., Lech, C.J., Heddi, B., Nicolas, A. and Phan, A.T. (2014) Structure and conformational dynamics of a stacked dimeric G-quadruplex formed by the human CEB1 minisatellite. *J Am Chem Soc*, **136**, 6297-6305.

10. Crnugelj, M., Sket, P. and Plavec, J. (2003) Small change in a G-rich sequence, a dramatic change in topology: new dimeric G-quadruplex folding motif with unique loop orientations. *J Am Chem Soc*, **125**, 7866-7871.

11. Nielsen, J.T., Arar, K. and Petersen, M. (2009) Solution structure of a locked nucleic acid modified quadruplex: introducing the V4 folding topology. *Angew Chem Int Ed Engl*, **48**, 3099-3103.

12. Kuryavyi, V. and Patel, D.J. (2010) Solution structure of a unique G-quadruplex scaffold adopted by a guanosine-rich human intronic sequence. *Structure*, **18**, 73-82.

13. Liu, Y., Lan, W., Wang, C. and Cao, C. (2018) A putative G-quadruplex structure in the proximal promoter of VEGFR-2 has implications for drug design to inhibit tumor angiogenesis. *J Biol Chem*, **293**, 8947-8955.

14. Wan, C., Fu, W., Jing, H. and Zhang, N. (2018) NMR solution structure of an asymmetric intermolecular leaped V-shape G-quadruplex: selective recognition of the d(G2NG3NG4) sequence motif by a short linear G-rich DNA probe. *Nucleic Acids Res*, **47**, 1544-1556.

15. Butovskaya, E., Heddi, B., Bakalar, B., Richter, S.N. and Phan, A.T. (2018) Major G-Quadruplex Form of HIV-1 LTR Reveals a (3 + 1) Folding Topology Containing a Stem-Loop. *J Am Chem Soc*, **140**, 13654-13662.

16. Marusic, M. and Plavec, J. (2019) Towards Understanding of Polymorphism of the G-rich Region of Human Papillomavirus Type 52. *Molecules*, **24**, 1294.

17. Haase, L., Dickerhoff, J. and Weisz, K. (2019) Sugar Puckering Drives G-Quadruplex Refolding: Implications for V-Shaped Loops. *Chem Eur J,* **25**, DOI: 10.1002/chem.201904044.

18. Truong, T.H.A., Winnerdy, F.R. and Phan, A.T. (2019) An Unprecedented Knot-like G-Quadruplex Peripheral Motif. *Angew Chem Int Ed Engl*, **58**, 13834-13839.
